# Quantitative mapping of mercury and selenium in mushroom fruit bodies with laser ablation–inductively coupled plasma-mass spectrometry

**DOI:** 10.1007/s00216-022-04240-y

**Published:** 2022-08-05

**Authors:** Simone Braeuer, Tom Van Helden, Thibaut Van Acker, Olivier Leroux, Dominique Van Der Straeten, Annemieke Verbeken, Jan Borovička, Frank Vanhaecke

**Affiliations:** 1 Atomic & Mass Spectrometry – A&MS research unit, Department of Chemistry, Ghent University, Campus Sterre, Krijgslaan 281 – S12, 9000 Ghent, Belgium; 2 Institute of Chemistry, University of Graz, Universitaetsplatz 1, 8010 Graz, Austria; 3 Laboratory of Functional Plant Biology, Department of Biology, Ghent University, K. L. Ledeganckstraat 35, 9000 Ghent, Belgium; 4 Research Group Mycology, Department of Biology, Ghent University, K. L. Ledeganckstraat 35, 9000 Ghent, Belgium; 5 Nuclear Physics Institute of the Czech Academy of Sciences, Hlavní 130, 25068 Husinec-Řež, Czech Republic; 6 Institute of Geology of the Czech Academy of Sciences, Rozvojová 269, 16500 Prague 6, Czech Republic

**Keywords:** Bioimaging, Heavy metal, Trace element, Macrofungi, LA-ICP-MS, Quantification

## Abstract

This work describes the development of a novel method for quantitative mapping of Hg and Se in mushroom fruit body tissues with laser ablation coupled to inductively coupled plasma-mass spectrometry (LA-ICP-MS). Different parameters of the protocol for preparation of the standards used for quantification via external calibration were assessed, e.g., the dissolution temperature of gelatin standards and the addition of chitosan and l-cysteine as additives to the gelatin-based calibration droplets to better match the sample matrix. While chitosan was not suited for this purpose, the presence of l-cysteine considerably improved the figures of merit of the calibration, leading to limits of detection of 0.006 and 0.3 µg g^−1^ for Hg and Se, respectively, at a pixel size of 20 × 20 µm. Further, an in-house reference material, ideally suited for the validation of the method for application to mushroom samples, was successfully prepared from a paste of *Boletus edulis*. The newly developed method was used to investigate the distribution of Hg and Se in tissue sections of five porcini mushroom individuals of three different species (*Boletus edulis*, *Boletus aereus*, and *Boletus pinophilus*) and one sample of a parasol mushroom (*Macrolepiota procera*)*.* For one sample, additional areas were ablated at higher spatial resolution, with a laser spot size down to 5 µm, which allows a detailed investigation of the spatial distribution of Hg and Se in mushrooms.

**Graphical abstract:**

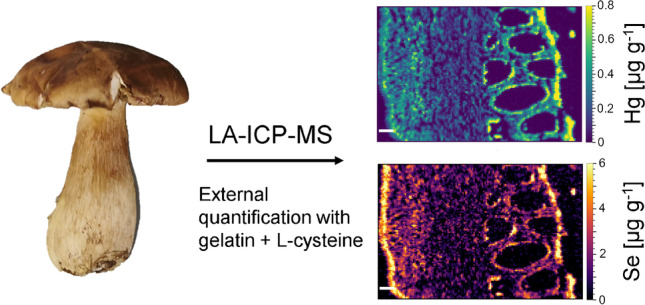

**Supplementary Information:**

The online version contains supplementary material available at 10.1007/s00216-022-04240-y.

## Introduction

Mercury is well known for its toxicity [1], with a tolerable weekly intake (TWI) of 4 µg kg^−1^ body weight for inorganic Hg [2]. High concentrations are mainly found in the marine environment, while terrestrial organisms usually contain much lower amounts of this heavy metal [2]. Also, in most macrofungi (mushrooms or macromycetes), the Hg concentration is < 0.5 µg g^−1^ dry mass (dm; around tenfold higher than for fresh samples, due to a water content of ca. 90%) [3]. It is usually assumed that mushrooms contain predominantly inorganic Hg (more than 90% of the total Hg), and only low amounts of the more hazardous methylmercury, although the available data is still limited [4]. However, some macrofungal species are known for their ability to accumulate higher concentrations of Hg in their fruit bodies, especially *Boletus edulis* and its close relatives *Boletus pinophilus*, *Boletus aereus*, and *Boletus reticulatus*, with up to ca. 20 µg g^−1^ dm [3, 5]. This is of special interest, because this group of species around *B. edulis*, famously known as porcini (IT), cèpes (FR), or Steinpilze (G), are some of the most sought and consumed type of wild-grown mushrooms worldwide. In addition to Hg, porcini mushrooms also accumulate Se, containing around 20 µg g^−1^ dm and more, as opposed to around 2 µg g^−1^ dm or even less in other macrofungal species [5, 6]. This parallel accumulation of Hg and Se is especially noteworthy, because Se is often discussed as a natural antagonist of Hg [7]. In addition, although Se is an essential trace element (recommended daily allowance, RDA, for adults: 55 µg) [8], it can also become toxic when administered in higher doses (tolerable upper limit, UL, for adults: 300 µg day^−1^), making it a double-edged sword [9].

Despite the potential health risks and/or benefits from high amounts of Hg and Se in these culinary delicacies, surprisingly few studies have been published that go beyond the reporting of bulk element concentrations in mushrooms and substrate soil. One notable exception is the recent work by Kavčič et al.(2019), who not only studied the bioavailability of Hg and the chemical species in which it is occurring in mushrooms, but also used laser ablation–inductively coupled plasma-mass spectrometry (LA-ICP-MS) and micro-X-ray fluorescence spectrometry (µ-XRF) to investigate the spatial distribution of Hg and Se in the fruit bodies [10], demonstrating the high potential of spatially resolved techniques to investigate the distribution of elements in mushrooms. However, the work reported in that paper is rather a first proof of concept, and details such as quality control, instrument settings, sample preparation, and calibration strategy were not addressed in detail. The same is also valid for the few other papers investigating the spatial distribution of elements in mushroom fruit bodies, namely As (and Ca) in *Lentinula edodes* and *Agaricus bisporus* [11] and in As-enriched *Agaricus bisporus* [12], determined by X-ray spectroscopic techniques, and again As in *Lentinula edodes* and *Pleurotus ostreatus* by LA-ICP-MS [13]. In all three works, only qualitative information is given, without quantification. Also worth mentioning is the comparatively early work by Turnau et al. (2001), who investigated the distribution of several major elements like P, S, and K, and of the potentially hazardous elements As, Cd, and Pb in mycorrhizal tips of *Suillus luteus* with proton-induced X-ray emission spectrometry (PIXE) [14]. The study demonstrates very clearly the importance of adequate sample preparation, and shows how chemical fixation negatively affects the quality of element maps of mycorrhizal thin sections.

When it comes to other biological matrices than fungi, the number of publications on spatially resolved measurements of Hg and/or Se is somewhat higher, and different techniques have been employed for this purpose, including LA-ICP-MS, X-ray spectroscopic techniques like (synchrotron-based, micro-) XRF [15], (nanoscale) secondary ion mass spectrometry (SIMS) [16], and laser-induced breakdown spectroscopy (LIBS) [17]. From these approaches, LA-ICP-MS offers excellent limits of detection (LODs, down to the ng g^−1^– low pg g^−1^ level, depending on the element and ablation parameters), multi-elemental capabilities, a relatively simple sample preparation, comparatively good robustness against matrix effects, and a wide linear dynamic range [18–20]. Although its spatial resolution in the low micrometer range (mostly around 5–50 µm) is not as high as some of the other techniques mentioned [15], it is sufficient for addressing many research questions, and thanks to improvements in the last decade, sub-micrometer resolution (roughly > 0.5 µm) can be achieved under the right circumstances, such as, e.g., high element concentration, optimized instrumental setup for fast washout, etc. [21].

Several papers report efforts to elucidate the spatial distribution of Hg with LA-ICP-MS using simple line scans on samples like hair [22], dorsal spine of fish [23], or turtle carapace [24], to reveal variations in exposure to this heavy metal over time. X-ray spectroscopic techniques have been employed to investigate Hg uptake and its 2-dimensional distribution in plants [25], fish [26], and whale brains [27]. LA-ICP-MS mapping experiments focusing on Hg have been reported as well, e.g., analyzing thin sections of plants [28, 29], fruit flies [30], glass eels [31], rodent tissues [32], eagle liver tissue [33], and human soft tissues [34]. In most cases, these experiments were carried out on samples with artificially elevated Hg concentrations, e.g., by adding Hg to the animal feed or plant substrate.

Regardless of the elements investigated, quantification is still challenging in LA-ICP-MS analysis [35]. In many cases, presenting only relative differences in signal intensity may be sufficient [36], but undoubtedly more valuable information can be gained through proper quantification with adequate quality control. Since potential elemental fractionation effects in LA-ICP-MS are matrix-dependent, this has to be accounted for during calibration [19]. A variety of different strategies has been proposed, including the preparation of pressed pellets from (certified) reference material [(C)RM] powders [22, 37], homogenized samples that are spiked with different concentrations of the analyte [32, 35], isotope dilution [20], determination of bulk concentrations of adjacent thin sections [38], and solution-based calibration via pneumatic nebulization [39]. Another popular approach is the use of pseudo-matrix-matched materials, like gelatin for animal tissues [40] or agarose for plant matrices [41], spiked with different concentrations of the elements of interest, as “matrix-matched” standards. Solutions of these materials are then spotted onto microscope slides, dried, and subjected to LA-ICP-MS analysis. However, this procedure can lead to pronounced inhomogeneities, sometimes referred to as “coffee stain” effects, where the element concentrations are higher at the edges of the droplet, or as “Marangoni” effects, where the concentrations are higher in the droplet’s center [42]. It has been shown that the homogeneity can be improved for some elements by drying gelatin droplets in an oven at elevated temperature under controlled conditions [42, 43]. Also, the preparation of gelatin or agarose samples in molds can lead to a more homogeneous distribution [41]. Another approach is the complete ablation of droplet standards, and the subsequent use of the absolute element quantities present in the droplets and the total integrated signal intensities to construct the calibration curves [44]. To avoid unnecessarily long measurement times, efforts are done to make such droplets as small as possible, which can be achieved with the help of low-volume pipette tips or, for even smaller volumes and automation of the process, micro-droplet spotters [45].

While reliable quantification in LA-ICP-MS is challenging for all elements, there are additional obstacles to be faced in the specific case of Hg. For example, Debeljak et al. pointed out unusually long washout times for Hg during LA-ICP-MS analysis, forcing them to perform spot analysis on a virtual grid of the sample surface and including 10 s waiting time after each spot [28]. However, in the 9 years since this publication, instrumentation and methods have improved significantly, and it is now possible to investigate the distribution of Hg and Se with an optimized setup at a pixel acquisition rate of 20 pixels per second [46]. Other concerns were raised regarding the quantification of Hg, since this element is especially prone to effects like evaporation or adsorption. According to Arnaudguilhem et al., addition of chloride or cysteine could not improve the stability of Hg in spin-coated dextran standards [47]. On the other hand, Niehoff et al. successfully added *meso*-2,3-dimercaptosuccinic acid (DMSA) as complexing agent for stabilization and used alkaline, ammonia-containing gelatin standards [30].

In the present work, an efficient method was developed for quantitative mapping of Hg and Se in mushroom fruit bodies via LA-ICP-MS with excellent limits of detection (LODs) and high spatial resolution (down to 5 µm). Traditional gelatin standards were compared to chitosan-containing ones, with the latter better mimicking a mushroom matrix. Chitosan is the deacylated and more soluble form of chitin [48], one of the main constituents of fungal cell walls [49]. Another approach was the addition of cysteine. With only around 30 mg kg^−1^ fresh mass [50], cysteine is one of the amino acids with the lowest abundance in mushrooms (in its free form), but can still serve as representative for the free amino acid content of mushrooms (around 1–100 g kg^−1^ dry mass) [49]. More importantly, due to its ability to bind Hg via its –SH function, it is widely used in analytical chemistry for the stabilization of Hg, for example in Hg speciation analysis via liquid chromatography coupled to ICP-MS [51]. Also, the influence of the temperature during the dissolution and drying of calibration standards was evaluated. For method validation, an in-house reference material was prepared from a mushroom powder. The final method was then applied to tissue sections of six different mushroom samples.

## Materials and methods

### Statement of human and animal rights

Not applicable.

### Chemicals and instruments

Ultrapure water (resistivity > 18.2 MΩ*cm) was used throughout the study. HNO_3_ and HCl were purchased from Fisher Chemicals and further purified via sub-boiling distillation. All other chemicals were purchased from common chemical suppliers and used without further purification. A detailed description of all chemicals, including purity and supplier, is provided as supplementary material, Online resource 1. The instruments used are also listed in more detail in Online resource 1, and are only described in a concise way here.

A Multiwave 7000 (Anton Paar, Graz, Austria) was used for pressurized, microwave-assisted acid digestion. Bulk element concentrations were determined via tandem ICP-MS (ICP-MS/MS), using an 8800 ICP-MS/MS unit (Agilent Technologies, Tokyo, Japan). For LA-ICP-MS experiments, an Iridia LA-unit (Teledyne Photon Machines, Bozeman, MT, USA), equipped with a 193-nm ArF* excimer-based nanosecond 1-kHz lasing system (MLase AG, Germering, Germany) and a cobalt ablation chamber with optimized cup-type ablation cell was coupled via an ARIS (aerosol rapid introduction system, Teledyne Photon Machines, Bozeman, MT, USA) [52] to an Agilent 7900 ICP-MS unit (Agilent Technologies, Tokyo, Japan).

### Determination of bulk concentrations

To determine bulk element concentrations in prepared standards, mushroom samples, and (C)RMs, the samples were digested with 14 mol L^−1^ HNO_3_ in polytetrafluoroethylene (PTFE) vessels, in a pressurized microwave oven (40 bar N_2_ loading pressure, heated up to 250 °C in 20 min and held there for another 20 min). The digests were diluted with ultra-pure water to 5% (v/v) HNO_3_, and 2% (v/v) HCl was added. The samples were then subjected to ICP-MS/MS analysis, operated in oxygen reaction mode. Settings and performance data can be found in Online resource 1, Table S1. Quantification was achieved through external calibration against pure element standards in 5% (v/v) HNO_3_ and 2% (v/v) HCl. The CRMs TORT-3 (lobster hepatopancreas, National Research Council NRC-CNRC, Ottawa, Canada) and DORM-4 (fish protein, NRC-CNRC) were used for quality control. Ge and Lu (5 µg L^−1^) were added to all solutions as an internal standard correcting for potential matrix effects and signal instability.

### LA-ICP-MS measurements

The LA settings are listed in Table 1, and were optimized for fast combined monitoring of Hg and Se [46]. The ICP-MS unit was operated in no-gas mode. The performance was checked daily while ablating NIST SRM 610 (trace elements in glass, National Institute for Standards and Technology, Gaithersburg, MD, USA), and the settings were tuned for high sensitivity, low laser-induced elemental fractionation, and low oxide ratios. The dosage (number of shots per position/degree of spot overlap) was set to ablate the whole standard or sample, which was tested on a separate gelatin droplet by visual inspection after consecutive single shots. The selected laser energy density was too low for achieving ablation of the glass microscope slide (verified by monitoring ^29^Si^+^, with SiO_2_ being the major component of the soda-lime-silica glass of the microscope slide), and thus, no signal contribution originating from co-ablation of the glass could occur under these conditions. The concept of selective ablation of biological tissue on a glass substrate by controlling the laser energy density of nanosecond 193-nm laser radiation has been described by Van Acker et al. [53]. Performance data can be found in Online resource 1, Table S2.

**Table 1:** LA-ICP-MS settings for measuring Hg and Se or Hg, Se, Cu, and Ag

Parameter	Hg and Se only	Hg, Se, Cu, and Ag
Energy density	1.5 J cm^−2^	1.0 J cm^−2^
He carrier gas flow rate	0.5 mL min^−1^	0.5 mL min^−1^
Spot size	20 µm	20 µm
Spot shape	Circle	Circle
Laser repetition rate	300 Hz	500 Hz
Dosage	15 shots position^−1^	25 shots position^−1^
Scan speed	400 µm s^−1^	400 µm s^−1^
Offset between lines	20 µm (= no gap)	20 µm (= no gap)
Integration times		
m/z 202 (Hg)	21 ms	15 ms
m/z 82 (Se)	21 ms	15 ms
m/z 107 (Ag)	–	4 ms
m/z 63 (Cu)	–	5 ms
Total scan cycle time*	50 ms	50 ms

*Sum of integration times + settling time.

### Calibration

Different matrices were evaluated for the production of matrix-matched calibration standards: (A) 10% (m/m) gelatin, (B) 1% (m/m) chitosan with additional gelatin (see next section), and (C) 10% (m/m) gelatin with 5 mmol L^−1^ l-cysteine. Standards were prepared by adding gelatin powder, water, and stock solutions of the selected elements into 1.5-mL microcentrifuge tubes. In addition to this, chitosan powder was added to obtain matrices of type B, and l-cysteine stock solution (100 mmol L^−1^ in 1% nitric acid) was added to obtain matrices of type C. In all cases, the solutions were homogenized using a vortex mixer and placed in a water bath at 55 °C for 1 h for complete dissolution. For the preparation of droplets, the solutions were used while they were still warm. Large droplets were spotted onto microscope glass slides with transfer pipette tips. In the case of extrasmall droplets (“micro-droplets”), the glass slides were placed on a microbalance (M3P, Sartorius, Goettingen, Germany) and droplets of 20–200 µg were spotted with a transfer pipette tip. All droplets and micro-droplets were left to dry at room temperature for 12 to max. 48 h, under a cover to protect them from contamination.

#### Chitosan

In a recent publication, Arnaudguilhem et al. decided against the use of chitosan for matrix-matched calibration standards because of a high background of some elements (Hg and Se were not mentioned) [47]. Additionally, the chitosan available in our lab was purified from crab shells and was only of “practical grade.” For these reasons, small aliquots of chitosan powder were digested, as described in “Determination of bulk concentrations,” and investigated for their Hg and Se concentrations with ICP-MS/MS.

For the preparation of droplets, mixtures with different chitosan concentrations were prepared, from 1 to 10% (m/m), in water and 0.5% acetic acid, and then put in a water bath for 2 h at 55 °C, with visual inspection every 15 min. Due to the poor solubility at higher concentrations, all further experiments were carried out with 1% of chitosan. Different concentrations of gelatin—between 1 and 10% (m/m)—were tested as an additive to chitosan, to evaluate whether this could improve the mixture’s characteristics.

#### Preparation conditions

It was evaluated whether the temperature and duration of heating for gelatin dissolution influenced the element concentrations, since Hg is known for its volatility. For three different matrices (10% gelatin, 1% chitosan + 4% gelatin, 10% gelatin + 5 mmol L^−1^ l-cysteine), six identical samples were prepared as described above, containing 25 µg g^−1^ of Se and 5 µg g^−1^ of Hg and Cu. To initiate dissolution, all samples were put in a water bath at 55 °C for 10 min. Afterwards, two replicates were left to stand at room temperature (ca. 21 °C), and two were kept in a water bath at 35 °C and two at 55° C. An aliquot was taken after the first 10 min from the “room temperature” samples, serving as control. After 60 and 120 min, an aliquot was taken from each sample, digested with nitric acid (see “Determination of bulk concentrations”), and the element concentrations were determined via ICP-MS/MS. For each matrix, a blank was prepared, kept at 55 °C for 120 min, and then digested and analyzed as well.

It has been shown that an elevated drying temperature can improve the homogeneity of the distribution of some elements within gelatin droplets [42]. To evaluate this for Hg and Se, 10% gelatin droplets containing Hg and Se (5 and 25 µg g^−1^, respectively) were prepared on three separate glass slides. One slide was left to dry at room temperature, another was dried in an oven at around 50 °C for 1 h, and the last one was dried in an oven at 90 °C, also for 1 h. They were then left to cool down in the oven overnight and subsequently investigated with LA-ICP-MS.

To evaluate the repeatability of the preparation procedures, five solutions of each matrix (10% gelatin, 1% chitosan + 4% gelatin, 10% gelatin + 5 mmol L^−1^ l-cysteine) were prepared, containing 5 µg g^−1^ Hg, 25 µg g^−1^ Se, and also 5 µg g^−1^ Cu and Ag. The latter two elements were added for “control” purposes and were selected because they are also occurring at high concentrations in mushrooms. Further, Ag^+^ has an m/z more or less between those of Se^+^ and Hg^+^, and Cu had already been investigated with LA-ICP-MS by the authors in the past [44]. From each solution, a micro-droplet was prepared and measured with LA-ICP-MS.

### Final method

Calibration standards were prepared with 10% gelatin + 5 mmol L^−1^ l-cysteine as matrix. For dissolution, all standard mixtures were put in a water bath at 55 °C for 1 h, after which micro-droplets were produced, as described above. The concentrations of the calibration standards were 0.0, 0.1, 0.5, 1.0, 5.0, and 10 µg g^−1^ for Hg and 0.0, 0.5, 2.5, 5.0, 25, and 50 µg g^−1^ for Se. In addition, five blank micro-droplets were prepared with the same matrix as the calibration. The limits of detection (LODs) and limits of quantification (LOQs) were calculated as 3 and 10 times the blanks’ standard deviation, respectively, divided by the slope of the calibration curve.

### Samples

#### Wild mushroom samples

Within this study, a total of six fruit bodies were investigated: two samples of *Boletus edulis*, two samples of *Boletus aereus*, one sample of *Boletus pinophilus*, and one of *Macrolepiota procera* (parasol mushroom—usually containing Hg concentrations similar to porcini mushrooms, but only around 1–3 µg g^−1^ dm of Se [54])*.* Detailed information on the samples can be found in Online resource 2, Table S3. All samples were collected from unpolluted areas, with presumably background Hg and Se concentrations in the soils they were growing on; gently cleaned with a brush and a damp cloth; and cut into two equivalent parts. One half was kept frozen (− 20 °C) until further processing for spatially resolved analysis. The other half was freeze-dried and ground to a fine powder with a rotary mill (1-mm titanium sieve). This was used for determining the bulk element concentrations. Two of the samples were divided into their main parts (stipe, cap context, and tubes) before grinding.

#### In-house mushroom reference material

Since no suitable biological CRM for quantitative mapping of Hg and Se was available, an in-house mushroom reference material (MuRM) was prepared. A freeze-dried and powdered sample of *B. edulis* was selected (available from an unpublished pilot experiment) and mixed with small amounts of water until it was a thick, homogeneous paste, with a composition of 57% dry mushroom and 43% water, and a density of 1.1 g cm^−3^. A ring was cut out of a microcentrifuge tube to serve as a mold. To facilitate handling, it was cut in two halves, which were then put back together with tape. The mold was placed on a microscope glass slide, filled tightly with the mushroom paste, and then closed with another glass slide. It was secured with tape and frozen (− 20 °C). Aliquots of the remaining mushroom paste were used for digestion and analysis with ICP-MS/MS (see “Determination of bulk concentrations”).

#### Preparation of tissue sections

**Fig. 1:**
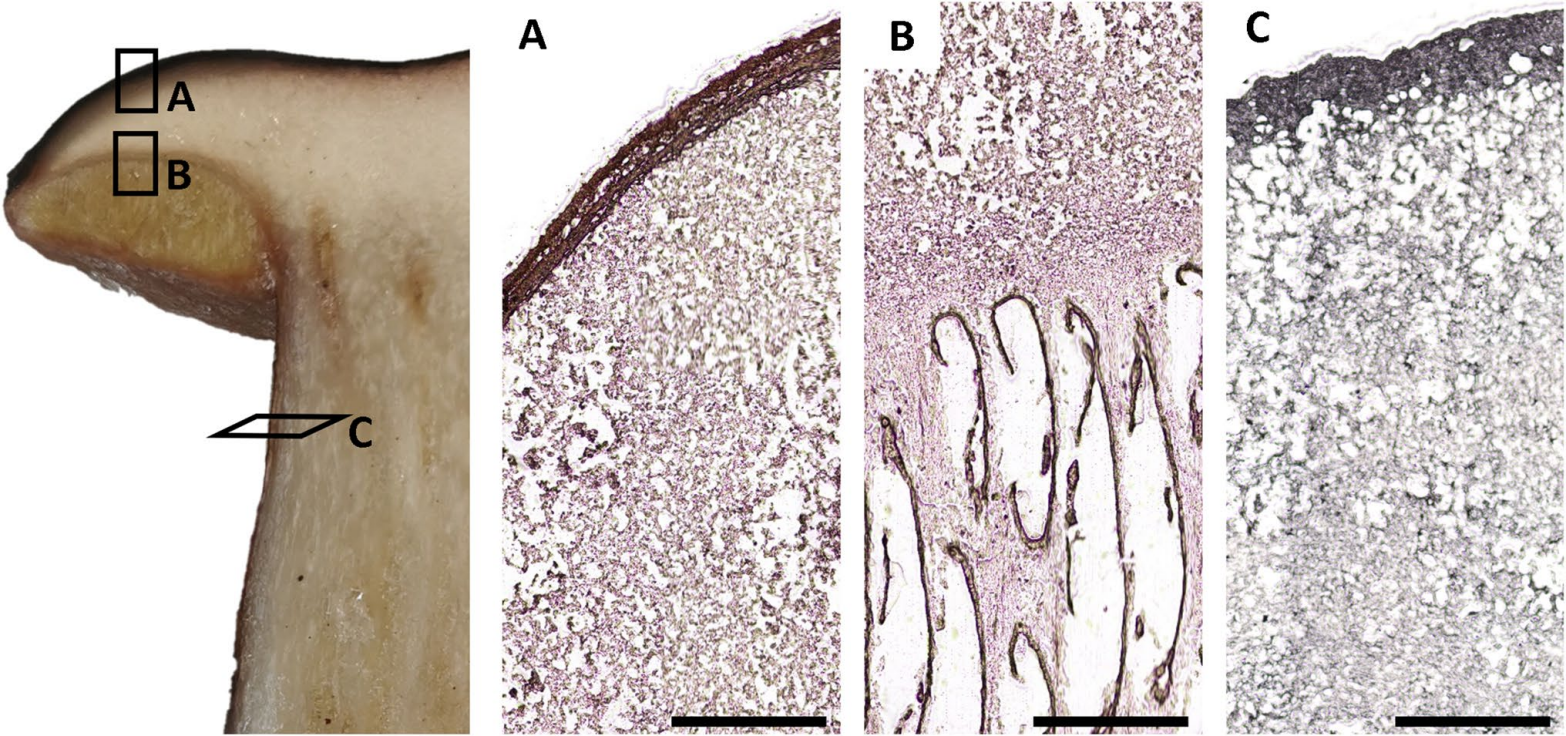
Cross section of a *Boletus* fruitbody collection with indication of sample locations and corresponding longitudinal (**A**, **B**) and transverse (**C**) tissue sections through the cap and stipe, respectively. **A**) Cap (peripheral and context tissue). **B**) Tubes and adjacent cap context tissue. **C**) Stipe (peripheral and context tissue). Black scale bars are 1 mm in length

Samples not exceeding 1 × 1 × 1 cm^3^ in volume were excised from the collected fruit bodies, as indicated in Fig. 1, from the cap, consisting of context (flesh, inner tissue) and pileipellis (peripheral tissue) or hymenophore (tubes or lamellae), indicated as areas A and B, respectively, in Fig. 1, and from the stipe, consisting of context (flesh, inner tissue) and stipitipellis (peripheral tissue), indicated as area C in Fig. 1.

Tissue-Plus O.C.T. medium was used to firmly fix the tissue blocks onto the sample holder by freezing at − 45 °C in a cryostat (Thermo Shandon FSE, Waltham, MA, USA). The thickness was set to 10 µm, and sections were cut at a chamber temperature of − 25 °C and collected on cryo-compatible adhesive tape. These were placed onto polylysine-coated slides with the sections facing upwards and stored in a slide box at 4 °C until analysis.

The same procedure was applied to the MuRM. In addition, further MuRM sections were cut and placed directly in glass digestion vessels. Since 10-µm sections were too brittle and could not be transferred quantitatively into the vessels, three vessels were filled with 15-µm-thick sections, and three with 20-µm-thick sections (five sections per vessel, to increase the total sample mass to around 7–10 mg and thus facilitate weighing and analysis). They were weighed, digested, and then analyzed for their bulk elemental composition with ICP-MS/MS (see also “Determination of bulk concentrations”).

#### Quantitative mapping of mushroom samples and MuRM

Microscope slides with mushroom or MuRM thin sections were mounted in the sample holder and placed in the ablation chamber. Regions of interest were selected with the help of the built-in camera, with a size of typically around 0.5–8 mm^2^, and then analyzed using the same instrument settings and data acquisition conditions as used for the calibration standards (Table 1). For the MuRM thin section, five replicates were measured, each with an area of 1.6 mm^2^. After every other mushroom sample, five lines of 1 mm length were ablated on a gelatin droplet standard containing 5 and 25 µg g^**−**1^ of Hg and Se, respectively, to correct for potential instrumental signal drift.

#### Higher spatial resolution mapping of mushroom samples

On the thin section of the stipe of sample POR-031, four consecutive areas were ablated with different laser beam diameters, namely 20, 15, 10, and 5 µm. The data obtained from this experiment was of qualitative nature only, as calibration was done at 20 µm only. The other settings were kept constant. With each spot size, an area of 900 × 450 µm was ablated.

### Data handling

**Fig. 2:**
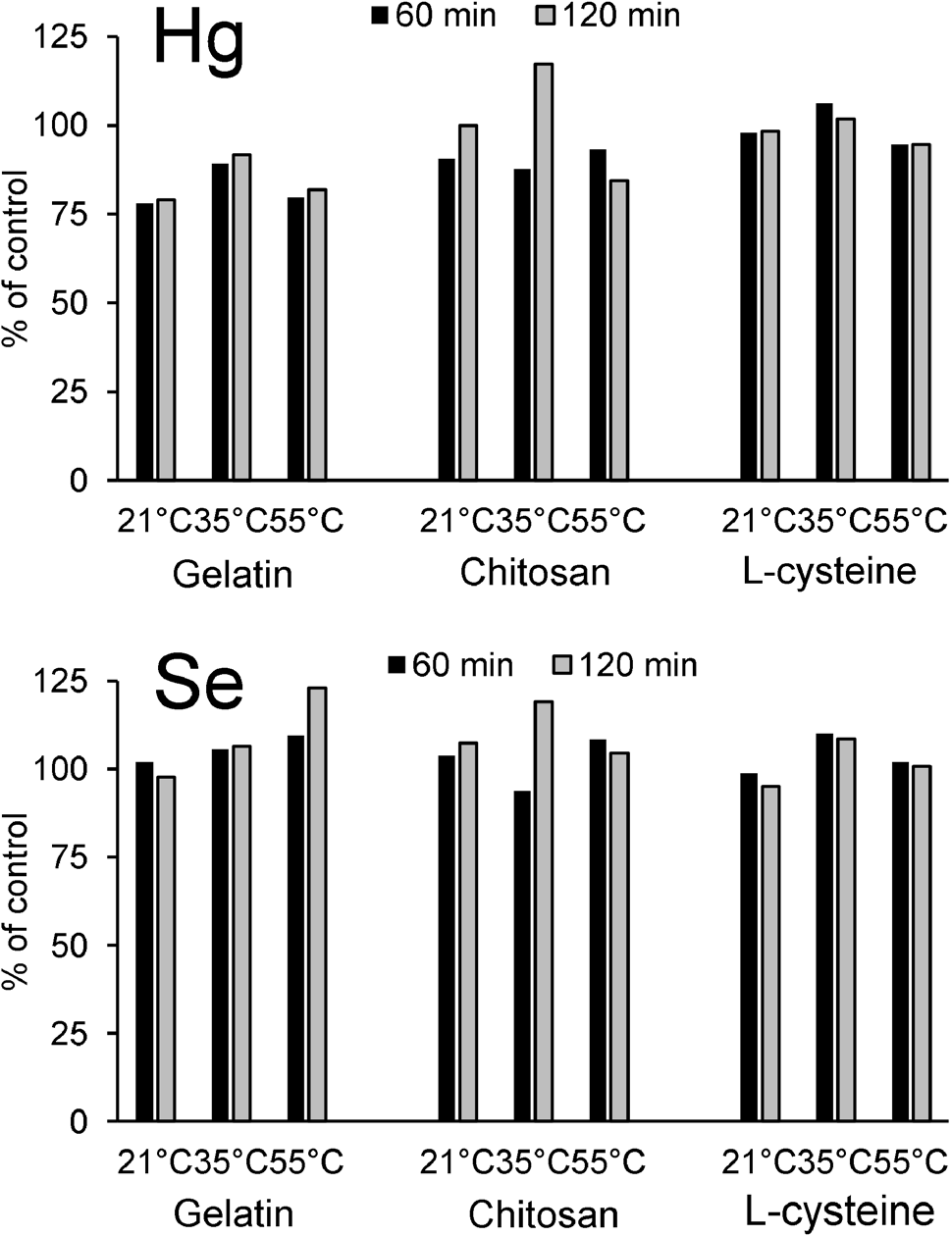
Recovery of Hg and Se (5 and 25 µg g^−1^, respectively) in droplets with three different matrices, 10% gelatin (“Gelatin”), 1% chitosan + 4% gelatin (“Chitosan”) and 10% gelatin + 5 mM L-cysteine (“L-Cysteine”) after 60 and 120 min at 3 different preparation temperatures (21° C*, 35° C*, 55° C). Data presented as mean of 2 replicates, relative to the control sample (10 min at 55° C, not shown). *First 10 min at 55° C, to enable the dissolution of gelatin

ICP-MS data were recorded with MassHunter v4.4 software, version C.01.04 (Agilent Technologies). HDIP software (Teledyne CETAC Technologies, v1.3.3.1091) was used to process LA-ICP-MS data: synchronization of laser log files and time-resolved ICP-MS data, background subtraction, splitting the data into 2D elemental maps, and drawing and integration of regions of interest (ROIs; note that the term used in HDIP is VOIs, for volumes of interest) within the element maps. Further calculations were done with Microsoft Excel 2016, which was also used for generating Fig. 2.

## Results and discussion

### Bulk concentrations of CRMs and chitosan powder

The results for the digested CRMs (analyzed via solution-based ICP-MS/MS) were in good agreement with the corresponding reference values, with levels of trueness between 88 and 108% (see also Table S4 in Online resource 2).

The Hg background in the chitosan powder was < 1 µg kg^−1^. The Se background was higher at around 90 µg kg^−1^, but still much lower than the Se calibration range aimed for, and was thus acceptable for our purpose.

### Calibration standards

#### Chitosan

The first attempts to prepare chitosan-containing solutions showed that a maximum of 1% chitosan could be dissolved in water or 0.5% acetic acid, even after 2 h of heating at 55 °C. From 2% chitosan onwards, it was not possible to obtain homogeneous solutions, and the mixtures were too viscous to prepare droplets. Increasing the amount of acidic acid was not possible, since this would have inhibited the gelation process. Thus, 1% chitosan was chosen for all chitosan-containing preparations. Next, different concentrations of gelatin were added to 1% chitosan, from 0 to 10%. It was observed that mixtures with 8% gelatin or more were extremely viscous and thus difficult to homogenize and pipet. Further, the center of the droplets looked different from the rest of the droplets, somewhat “disturbed,” which was most pronounced with low amounts of gelatin (0–2%; see microscope image in Fig. S1, Online resource 1). For these practical reasons, it was decided to use 1% chitosan with 4% gelatin henceforth.

#### Dissolution conditions

The influence of the preparation temperature (21 °C, 35 °C, and 55 °C) and the preparation time (10, 60, and 120 min) on the Hg, Se, and Cu (as control) concentrations in three different matrices was evaluated. For each time and temperature, duplicate samples were investigated for their bulk element concentrations. The mean values of these duplicates are presented in Fig. 2. All individual results can be found in Online resource 2, Table S5. The overall median deviation between the two replicates was 4.3% (taking into account Hg, Se, and Cu), but there were differences between the three different matrices: 6.1% for gelatin, 3.8% for l-cysteine, and 4.7% for chitosan. The highest variability for Hg and also Se could be observed for the chitosan samples, although without a clear trend, with a recovery varying between 84 and 117% compared to that for the control sample, in the case of Hg. The results for the gelatin and l-cysteine preparations were more constant. However, the Hg concentrations in the gelatin samples were only 78–92% of that of the control sample (taken after 10-min dissolution at 55 °C), while they were 95–106% in the l-cysteine samples. For Se, the recoveries in the l-cysteine preparations were 95–110% of that of the control sample. In the gelatin samples, they were between 98 and 123%. The concentrations in the blanks (120 min at 55 °C) were less than 1% of the standards. Overall, no apparent influence of the temperature or duration of the dissolution step on the element concentrations could be detected. On the other hand, the repeatability seemed to be strongly dependent on the matrix, and this was therefore investigated in a next step.

#### Repeatability

For each matrix (10% gelatin, 1% chitosan + 4% gelatin, 10% gelatin + 5 mmol L^−1^ l-cysteine), five micro-droplets with 5 µg g^−1^ Hg, Cu, and Ag, and 25 µg g^−1^ Se, were completely ablated, and the integrated signals of ^202^Hg^+^, ^82^Se^+^, ^63^Cu^+^, and ^107^Ag^+^ (in the case of chitosan, only ^202^Hg^+^ and ^82^Se^+^) were recorded. They were normalized to the mass of the individual droplets and then compared with each other. The relative standard deviations (RSDs) for these replicates are shown in Table 2. For the gelatin matrix, the RSDs were as high as 23 and 28% for Hg and Se, values were similar for the chitosan matrix (20 and 21%, respectively), but much better RSDs could be achieved for the target elements Hg and Se in the l-cysteine matrix, namely 2.3 and 3.9%. Also, for Cu and Ag, which were included for comparison, the RSDs were better in the l-cysteine preparations than in the gelatin ones, but still around 10% (instead of 19%).

**Table 2:** Relative standard deviations (in % of the mean signal per mg droplet, *n* = 5) for the analytes in micro-droplets made of 10% gelatin (“Gelatin”), 1% chitosan + 4% gelatin (“Chitosan”), and 10% gelatin + 5 mmol L^−1^ l-cysteine (“l-cysteine”)

	Hg	Se	Cu	Ag
Gelatin	23	28	19	19
Chitosan	20	21	n.a	n.a
l-Cysteine	2.3	3.9	9.6	11.3

*n.a.* not analyzed.

**Fig. 3:**
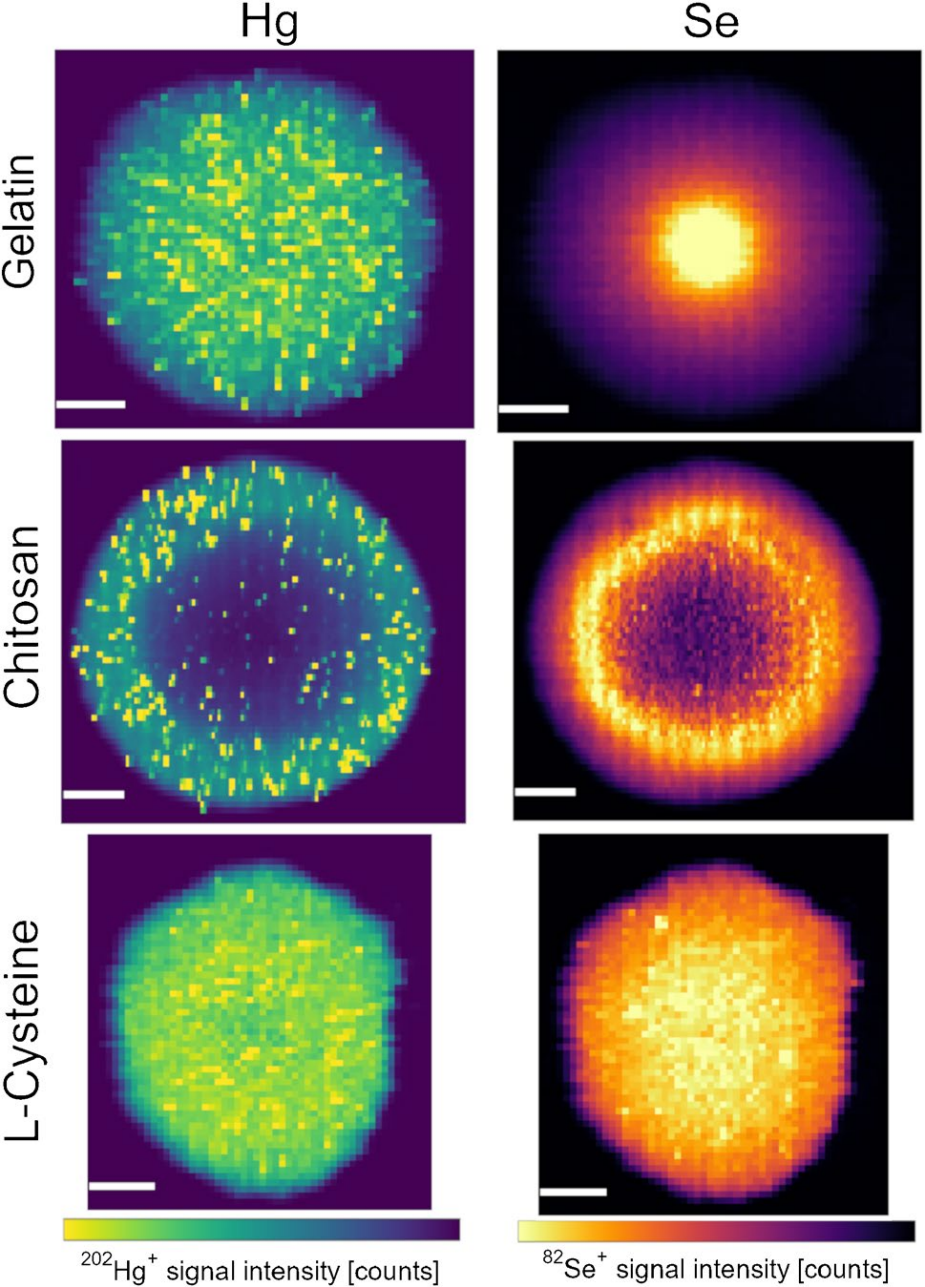
Hg and Se distribution in micro-droplets of 10% gelatin (“Gelatin”), 1% chitosan + 4% gelatin (“Chitosan”) and 10% gelatin + 5 mM L-cysteine (“L-cysteine”), containing 5 µg g^−1^ Hg and 25 µg g^−1^ Se (and 5 µg g^−1^ Cu and Ag, shown in Fig. S2, Online Resource 1). The minimum–maximum signal intensities for the color bars are 0–2*10^4^, 0–1.5*10^4^ and 0–2.5*10^4^ counts for ^202^Hg^+^ and 0–8*10^3^, 0–4*10^3^ and 0–4.5*10^3^ counts for ^82^Se^+^, from top to bottom, respectively. All white scale bars are 200 µm

Furthermore, LA-ICP-MS mapping demonstrated that Hg and Se were distributed extremely unevenly within the chitosan-containing micro-droplets (Fig. 3), reflecting to some extent the macroscopically distinct center, which was already mentioned above (see also Fig. S1, Online resource 1). For Hg, a ring of increased intensity could be observed on top of random hot spots. In the case of Se, a low-intensity center and edge, and a high-intensity ring in-between, were found. As shown in Fig. 3, the distribution of Hg in the gelatin micro-droplets was slightly better than in chitosan, but many small “hot spots” were scattered over the entire droplet. For Se, the Marangoni effect was observed in the gelatin micro-droplets, with high intensities in the droplet center. Addition of l-cysteine led to a significant improvement, with a much more homogeneous distribution of both Hg and Se over the entire micro-droplet. It seems that the physical and/or chemical properties of the droplets are significantly altered through the addition of l-cysteine. We hypothesize that l-cysteine could serve as cross-linker or complexing agent not only for Hg, but also for Se, facilitating a homogeneous dissolution and distribution. It might also be possible that the drying process is proceeding more homogeneously. This should be investigated further in the future.

Although addition of chitosan to the calibration droplets would have matched the matrix of mushrooms much better than gelatin alone, it was decided not to use this compound. Not only was the distribution within these droplets extremely inhomogeneous, but also the precision was sub-par in all experiments. This can be partly attributed to the poor solubility, which made the preparation of solutions much more tedious and prone to errors.

##### Drying conditions

Gelatin droplets containing 5 µg g^−1^ of Hg and 25 µg g^−1^ of Se were dried at three different temperatures to see whether this could improve the homogeneity of the distribution of the two elements within a droplet, as was already reported for other elements [42]. The corresponding LA-ICP-MS images can be found in Online resource 1, Fig. S3. In the case of Hg, elevating the drying temperature led to an increase in the occurrence of hot spots and thus decreased the homogeneity. For Se, the drying temperature had no effect on this distribution. The signal intensities remained higher in the center of the droplets, and gradually decreased towards the edges of the droplets.

Overall, the results also showed that gelatin alone is not the ideal matrix for calibration standards of Hg and Se, but that adding l-cysteine improves the repeatability and the homogeneity within micro-droplets considerably, which cannot be realized by drying of droplets at elevated temperatures. Thus, micro-droplets for quantifying Hg and Se with LA-ICP-MS were prepared from 10% gelatin with 5 mmol L^−1^ l-cysteine. It should also be noted that the effect of l-cysteine addition was less pronounced for Cu and Ag, which were included for comparison, and thus this cannot be regarded as a universal recipe. Indeed, the optimal calibration matrix and preparation conditions can be different from one element to the next, and therefore have to be explored for each element individually.

### Calibration

The results for the final calibration with 10% gelatin + 5 mmol L^−1^ l-cysteine in the ranges 0.1–10 and 0.5–50 µg g^−1^ (plus calibration blank; see also Fig. S4 and S5 in Online resource 1 for the visualization) for Hg and Se, respectively, were fit for purpose as presented in Table 3. LOD and LOQ were calculated as 3 and 10 times the standard deviation based on 5 individual blank micro-droplets. For Hg, the LOD of 0.006 µg g^−1^ is much better than previously reported values, which typically range between 0.04 and 4.9 µg g^−1^ [28, 30, 32]. For Se, the LOD is much higher at 0.3 µg g^−1^. In older literature data, however, the LOD for Se is sometimes even higher than that [39]. But, a much lower LOD has recently been published (0.03 µg g^−1^), which was achieved by employing ICP-MS/MS and addition of methanol to the aerosol [55]. Combining this strategy with our calibration method is an interesting option for future studies. Although LODs in LA-ICP-MS are strongly dependent on the type and amount of material ablated and the instrument settings, rough comparisons between methods with similar settings (e.g., spot size and energy density) can still be useful.

**Table 3:** Figures of merit of the Hg and Se calibration (0.1–10 and 0.5–50 µg g^−1^, respectively). The repeatability can be found in Table 2

	LOD [µg g^−1^]	LOQ [µg g^−1^]	Linearity (*R*^2^)
Hg	0.006	0.02	0.9994
Se	0.3	1	0.9979

### Reference mushroom homogenate

To validate the calibration, an in-house reference material, MuRM, prepared from a homogenized sample of *B. edulis*, was analyzed. Since the natural Hg and Se concentrations were already sufficiently high, no artificial Hg and Se had to be added during the production of the MuRM, which makes the material perfectly suited for method validation. To evaluate if the sectioning process influenced the element concentrations, thin sections and bulk material were digested and subjected to bulk analysis via ICP-MS/MS. Since it was physically not possible to do this with 10-µm-thick sections (the thickness that was used for LA-ICP-MS experiments), samples with two different thicknesses were prepared and compared with each other. As can be seen in Table 4, the Hg concentrations were indeed lower in the thin sections than in the bulk material. It is possible that part of the volatile Hg is released during the thin sectioning process, or shortly after. This also highlights the importance of determining the reference element concentrations in thin sections rather than in bulk material for better matching to the actual samples. It further indicates that quantitative data from the other investigated samples should be regarded with caution, since a thorough evaluation of the stability of Hg and Se in the fruit body thin sections was beyond the scope of this study. However, no difference could be seen between 15- and 20-µm-thin sections, which indicates that small differences in thickness do not significantly impact the Hg concentration. For Se, the recovery from the digested thin sections was very good, with 96 and 97% for 15- and 20-µm-thin sections, respectively. However, the concentration in the ablated MuRM was only 70 ± 1% of the bulk concentration, which is not ideal and leaves room for improvement. It can be speculated that this could be due to the probably different chemical forms in which Se is present in natural mushrooms and in pure standards, which could show, e.g., a different transport behavior (e.g., transport in particulate vs. in gaseous form) and/or a different degree of ionization in the ICP. In addition, it is known that Se exhibits a strong carbon enhancement effect in ICP-MS measurements, resulting in an increase of the recorded Se signal intensity with increasing carbon content in the plasma [56]. Because of different tissue thicknesses, it is likely that the amount of ablated carbon is higher for the gelatin standards than for the MuRM thin section. To verify this in a future study, an approach similar to the one recently reported by Billimoria et al. could be used, where methanol—or a carbon-containing gas like CH_4_ or CO_2_—is added to the gas stream [55]. However, the introduction of an additional gas stream will likely negatively influence the single pulse responses, necessitating slower measurement settings.

**Table 4:** Results for the in-house RM, MuRM, presented as mean ± standard deviation: element concentrations and recovery from digests of bulk material (*n* = 3) and thin sections (*n* = 3), as well as in ablated segments of a thin section (*n* = 5). The recovery from the digested thin sections is relative to the bulk concentrations, and the recovery from the ablated material is relative to the digested 15-µm-thin sections

	Concentration		Recovery	
	Hg [µg g^−1^]	Se [µg g^−1^]	Hg [%]	Se [%]
Bulk—digested	6.19 ± 0.43	11.51 ± 0.53		
15-µm-thin sections—digested	4.27 ± 0.22	11.06 ± 0.71	69 ± 4	96 ± 6
20-µm-thin sections—digested	4.34 ± 0.25	11.13 ± 0.42	70 ± 4	97 ± 4
10-µm-thin section—ablated	4.36 ± 0.08	7.78 ± 0.15	102 ± 2	70 ± 1

Besides similarity to the sample matrix and roughly matching analyte concentrations, another requirement for RMs intended for LA-ICP-MS is a homogeneous distribution of the analytes. The RSDs of the mean values of five, 1.6-mm^2^ large, ablated consecutive segments of the MuRM were 1.8 and 1.9% for Hg and Se, respectively, proving the homogeneous distribution of the analytes in the material. This can also be seen in the Hg and Se images of the MuRM, Fig. S6, in Online resource 1.

### Quantitative mapping of mushrooms

**Fig. 4:**
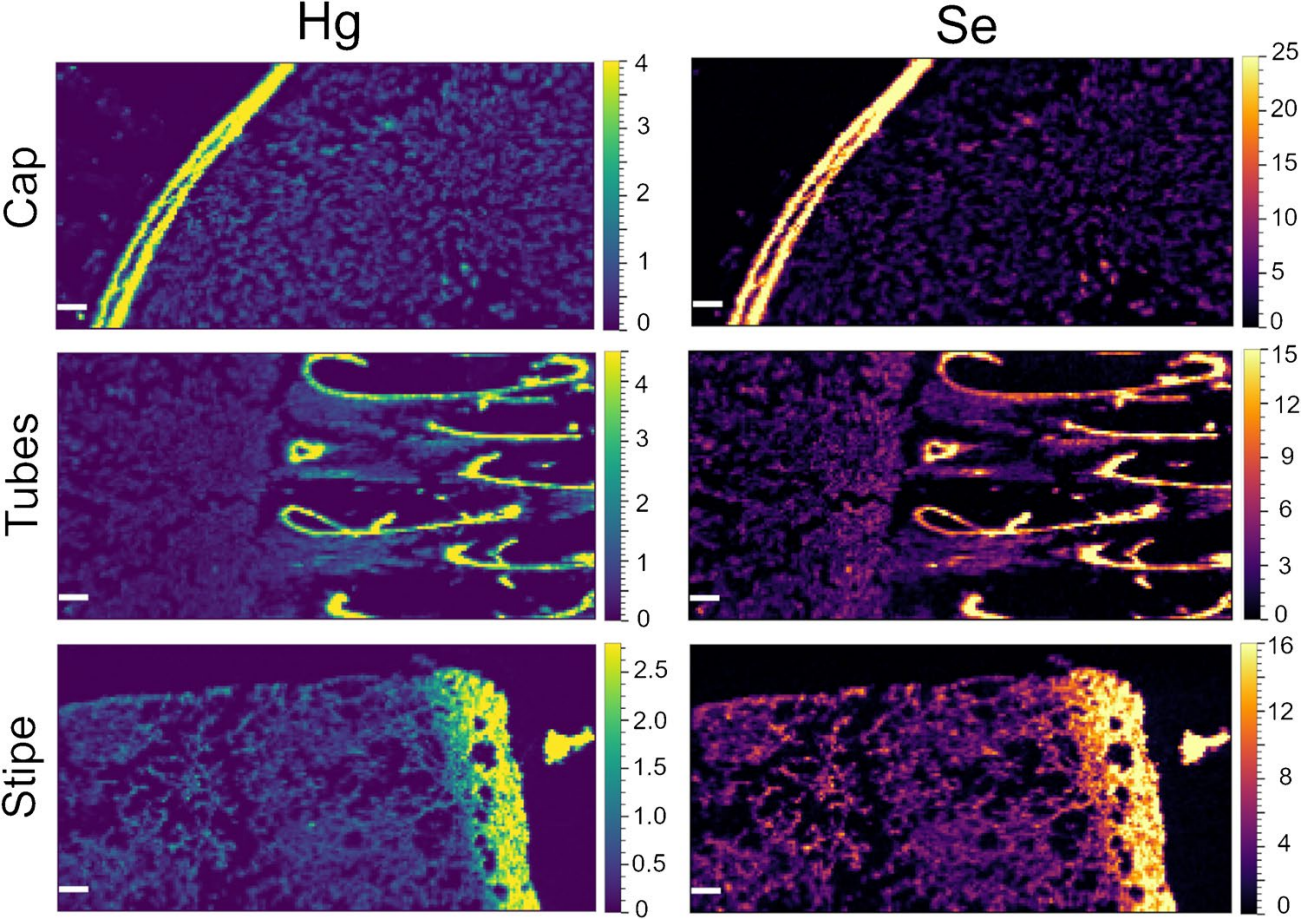
Quantitative distribution of Hg (left) and Se (right) in 4 × 2 mm areas of thin sections of sample POR-031 (*B. pinophilus*), color scales in µg g^−1^. Top: Cap (peripheral tissue on the left side, similar to location A in Fig. 1). Middle: Tubes (incl. adjacent cap tissue on the left side, similar to location B in Fig. 1). Bottom: Stipe (peripheral tissue on the right side, similar to location C in Fig. 1). All white scale bars are 200 µm

The developed and validated method was applied to thin sections of five porcini fruit body samples (*B. edulis*, *B. aereus*, *B. pinophilus*) and one parasol mushroom (*M. procera*). Maps for Hg and Se are shown for thin sections of the cap, tubes, and stipe of one mushroom sample in Fig. 4. All other Hg and Se images can be found in Online resource 1, Fig. S7 – S12. In all cases, the highest Hg and Se concentrations were found in the peripheral tissue of the stipe and cap. In some samples, this is accompanied by a gradual decrease towards the fruit body center, on a length of roughly 400–800 µm (e.g., the stipe sample in Fig. 4), whereas in other samples, there is a sharp cut, from high to low concentrations from the peripheral to the inner tissue (e.g., the cap sample in Fig. 4). In some cases, the high-concentrated region gets as thin as 40 µm (e.g., the stipe section in Fig. S7). However, it should be kept in mind that higher signal intensities could also result in part from a slightly higher local tissue density (e.g., because more mass is ablated). To evaluate this, theoretically, a matrix element like carbon could be monitored as internal standard and indicator of the amount of material ablated, but it has been shown that carbon is not well suited as internal standard [57], and our method was optimized for high-sensitivity measurements of Hg and Se, which would have been compromised to some extent by the addition of another element to the method.

The Hg and Se concentrations within the ablated sections and in individual regions of interest (ROIs) were calculated using HDIP, and are listed in full in Online resource 2, Table S6. The Hg concentrations ranged from 0.07 to 4.45 µg g^−1^, and the Se concentrations from 0.19 to 26.2 µg g^−1^. The lowest Se values were obtained for the sections of the *M. procera* sample, which is not surprising, since this fungal species is known not to accumulate Se to the same degree as porcini mushrooms, where the lowest Se concentrations were around 0.9 µg g^−1^. The Hg and Se values of the individual regions were 48–970% and 35–810% of the wet (or fresh) bulk concentrations. Given that there can already be striking differences between the various anatomical parts (i.e., cap, stipe, hymenophore), these large deviations are not surprising. The concentrations in the peripheral areas were 2.0–6.0 times higher than in the adjacent context tissue, as can be seen from the summary in Table 5. This enrichment factor was similar for Hg and Se in the same thin section, and also for the corresponding cap and stipe thin sections of one fruit body. An exception was sample POR-031, where the concentrations in the peripheral tissue of the cap were more elevated than in the peripheral tissue of the stipe. Further, Hg and Se are also significantly enriched in the lining of the tubes (hymenium), as can be seen in Fig. 4. Investigating the distribution of Hg and Se in spores of porcini mushrooms will thus be an interesting task for a future study.

**Table 5:** Enrichment factors of fruit body peripheral tissue compared to the adjacent context tissue for cap and stipe of all mushroom samples investigated (concentration in peripheral tissue divided by concentration in context tissue)

Sample ID	Species	Hg cap	Hg stipe	Se cap	Se stipe
POR-025	*B. aereus*	n.a	4.9	n.a	3.9
POR-026	*B. aereus*	2.5	2.5	2.0	2.2
POR-031	*B. pinophilus*	6.0	4.4	5.8	2.9
POR-070	*B. edulis*	n.a	3.2	n.a	3.7
STM-394	*B. edulis*	4.2	4.5	3.6	4.0
POR-067	*M. procera*	2.3	2.4	2.0	2.0

*n.a.* not available.

Although these differences between peripheral tissue (pellis) and inner tissue (context) are noteworthy, our results also show that they are not extreme. From a consumer’s point of view, the ingested amount of Hg will not be significantly lowered by “peeling” the fruit bodies, since it only accounts for a low percentage of the mushroom’s mass.

Overall, when comparing the TWI for inorganic Hg with the bulk Hg concentrations of the samples investigated, it would be safe for an average person with a body weight of 65 kg to consume between 0.3 and 2.6 kg of fresh mushrooms per week, on a regular basis. Interestingly, the situation is similar for Se: between 0.6 and 2.2 kg of porcini mushrooms could be eaten per week without exceeding the UL (not taking into account the one sample of *Macrolepiota procera*, which does not accumulate Se). On the other hand, already 15–60 g of fresh porcini mushrooms would be sufficient to fulfill the RDA. For the one sample of *M. procera*, 210 g would be necessary to reach the RDA. These values can also be found in Online resource 2, Table S3.

**Fig. 5:**
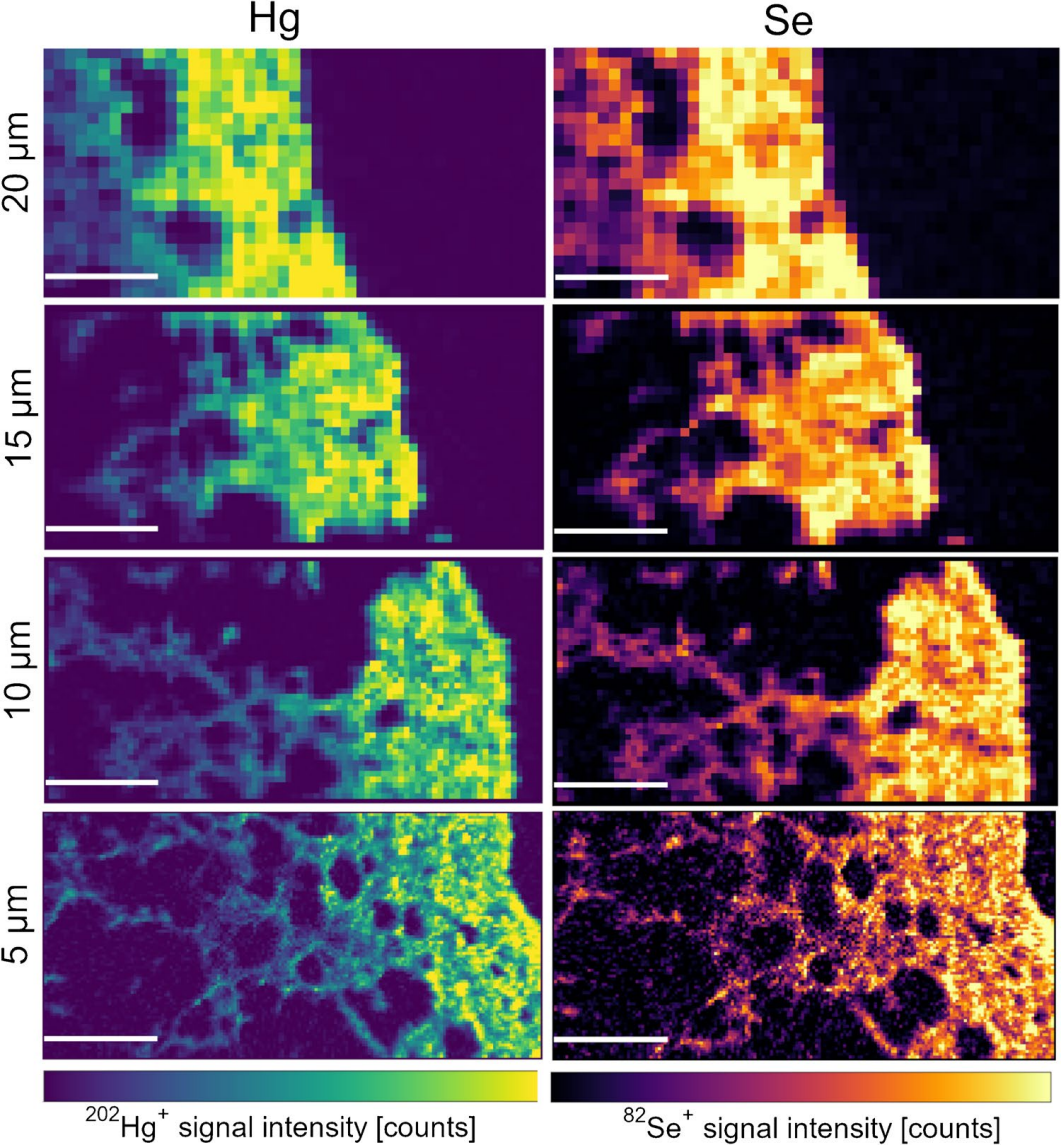
Hg (left) and Se (right) distribution in 0.9 × 0.45 mm areas of thin sections of the stipe of POR-031 (*B. pinophilus*, similar region as location C in Fig. 1) with decreasing spot size diameter (top to bottom: 20 – 15 – 10 – 5 µm). The peripheral tissue is on the right side of the images. The minimum–maximum signal intensities for the color bars are 0–1300, 0–1000, 0–500 and 0–120 counts for ^202^Hg^+^ and 0–600, 0–450, 0–200 and 0–50 counts for ^82^Se^+^, from top to bottom, respectively. All white scale bars are 200 µm

Finally, since the concentrations in the mushroom thin sections were sufficiently high to allow a further decrease of the laser spot size, four subsequent segments with decreasing spot sizes were ablated on one thin section, namely 20, 15, 10, and 5 µm. This was only intended as a proof of concept for future research, and was thus only done on a qualitative basis. As can be seen in Fig. 5, even with a pixel size of 5 × 5 µm, the signal intensities are still sufficiently high to get defined images, although there is already considerable noise in the case of Se. At this high spatial resolution, much finer structures can be distinguished. An overlay of the Hg and the Se maps suggests colocalization, as would be required for an antagonistic effect. Further investigation at even higher resolution could approve or disprove this hypothesis. The measurement of 0.4-mm^2^-large sections, as shown in Fig. 5 with the smallest spot size of 5 µm diameter, takes around 15 min only, which makes the method suitable for the investigation of large areas with a high spatial resolution in a reasonable amount of time.

## Conclusion and outlook

A method for fast quantitative mapping of Hg and Se in mushroom fruit bodies via LA-ICP-MS was developed. l-Cysteine was added to gelatin-based droplet standards, which improved the distribution and stability of the elements in the droplets. An in-house reference material was prepared from a mushroom paste and successfully used for validation of the calibration strategy. The analytical methodology was applied to cryo-sections of fruit bodies of five porcini mushrooms and one parasol mushroom. It was demonstrated that the Hg and Se concentrations were 2–6 times higher in the peripheral tissue of caps and stipes than in the adjacent context tissue, using a laser spot size of 20 µm diameter. By further decreasing the spot size to 5 µm for one sample, more details of the Hg and Se distributions could be revealed, and much finer structures could be distinguished. This clearly shows the potential for future studies, where the distribution of various elements in mushrooms could be investigated with even higher spatial resolution, provided that the concentrations are high enough. Such experiments will be necessary to unambiguously reveal a possible colocalization of Hg and Se at the (sub)cellular level, to further investigate the postulated Hg-Se antagonism.

## Supplementary Information

Below is the link to the electronic supplementary material.

Supplementary file1 Online Resource 1: Additional information on instruments, chemicals, instrument settings and performance (Table S1 and S2), as well as Fig. S1-S12. (DOCX 6.32 MB)

Supplementary file2 Online Resource 2: Excel file, containing the Supplementary Table S3-S6. (XLSX 38.7 KB)

## Data Availability

Most data generated during this study are included in the article and its supplementary online resources. Individual raw data is stored on the servers of Ghent University and can be obtained from the authors upon request.
