# Supplementary material for: Quantitative mapping of mercury and selenium in mushroom fruit bodies with laser ablation–inductively coupled plasma-mass spectrometry
Source: Anal Bioanal Chem. 2022 Aug 5;414(25):7517–30. doi: 10.1007/s00216-022-04240-y (PMC9482896; doi:10.1007/s00216-022-04240-y)
Supplement: Supplementary file 1 — Supplementary file1 Online Resource 1: Additional information on instruments, chemicals, instrument settings and performance (Table S1 and S2), as well as Fig. S1-S12. (DOCX 6.32 MB) [file 216_2022_4240_MOESM1_ESM.docx]

Online Resource 1

Quantitative mapping of mercury and selenium in mushroom fruit bodies with laser ablation - inductively coupled plasma-mass spectrometry

Simone Braeuer^a,b*^ (ORCID: 0000-0002-0975-9051), Tom van Helden^a^ (ORCID: 0000-0001-8728-7849), Thibaut Van Acker^a^ (ORCID: 0000-0002-0649-7228), Olivier Leroux^c^ (ORCID: 0000-0001-9723-5604), Dominique Van Der Straeten^c^ (ORCID: 0000-0002-7755-1420), Annemieke Verbeken^d^ (ORCID: 0000-0002-6266-3091), Jan Borovička^e,f^ (ORCID: 0000-0003-3966-558X), and Frank Vanhaecke^a^ (ORCID: 0000-0002-1884-3853)

^a^ Atomic & Mass Spectrometry – A&MS research unit, Department of Chemistry, Ghent University, Campus Sterre, Krijgslaan 281 – S12, 9000 Ghent, Belgium

^b^ Institute of Chemistry, University of Graz, Universitaetsplatz 1, 8010 Graz, Austria

^c^ Laboratory of Functional Plant Biology, Department of Biology, Ghent University, K.L. Ledeganckstraat 35, 9000 Ghent, Belgium

^d^ Research Group Mycology, Department of Biology, Ghent University, K.L. Ledeganckstraat 35, 9000 Ghent, Belgium

^e^ Nuclear Physics Institute of the Czech Academy of Sciences, Hlavní 130, 25068 Husinec-Řež, Czech Republic

^f^ Institute of Geology of the Czech Academy of Sciences, Rozvojová 269, 16500 Prague 6, Czech Republic

*Corresponding author. E-mail address: [simone.braeuer@uni-graz.at](mailto:simone.braeuer@uni-graz.at)

**Contents**

[1. Chemicals, materials and instruments 2](#_Toc100244930)

[2. ICP-MS/MS and LA-ICP-MS measurements 3](#_Toc100244931)

[3. Optimization of preparation procedures for calibration standards 4](#_Toc100244932)

[4. Hg and Se images of mushroom fruitbody samples 8](#_Toc100244933)

# Chemicals, materials and instruments

## Chemicals

- Ultrapure water, resistivity ≥18.2 MΩ*cm at 25 °C, Direct-Q3, Millipore, Molsheim, France
- 14 mol L^-1^ nitric acid, trace metal analysis grade, PrimarPlus, Fisher Chemicals, UK, further purified via sub-boiling distillation in a Savillex DST-4000 acid purification system, Savillex Corporation, USA.
- 12 mol L^-1^ hydrochloric acid, trace metal analysis grade, PrimarPlus, Fisher Chemicals, UK, further purified via sub-boiling distillation in a Savillex DST-4000 acid purification system, Savillex Corporation, USA.
- Mercury standard solution, 1000 mg L^-1^, Mercury (II) nitrate in HNO_3_ 0.5 mol/L, Merck, Darmstadt, Germany
- Selenium standard solution, 1000 µg mL^-1^, (Plasma HIQU), 2-5% HNO_3_, Chem-Lab NV, Zedelgem, Belgium
- Copper standard solution, 1000 µg mL^-1^, (Plasma HIQU), 2-5% HNO_3_, Chem-Lab NV, Zedelgem, Belgium
- Silver standard solution, 1000 µg mL^-1^ (Plasma HIQU), 2-5% HNO_3_, Chem-Lab NV, Zedelgem, Belgium
- Germanium, 999 ± 3 µg mL^-1^, traces. HF, tr. HNO_3_, inorganic Ventures, Christiansburg, Virginia, USA
- Lutetium, AA-Standard solution, 1000 µg mL^-1^ Lu in 5% HNO_3_, Alfa Aesar GmbH & Co KG, Karlsruhe, Germany
- Gelatin, Ph. Eur. Powder, VWR, Leuven, Belgium
- Chitosan, from crab shells, practical grade, Sigma-Aldrich, Steinheim, Germany
- L-Cysteine, Assay 97%, Sigma-Aldrich, Steinheim, Germany
- Acetic acid glacial, for analysis, ACS-ISO, Panreac Quimica SA, Montcada i Reixac, Spain
- Tissue-Plus O.C.T. medium, Fisher Scientific, Waltham, USA
- Cryo-compatible adhesive tape, Cryofilm 2C, Section-Lab, Hiroshima, Japan

## Instruments

- Rotary mill: ZM200, 1 mm titanium sieve, Retsch GmbH, Haan, Germany)
- Microwave-assisted digestion system: Multiwave 7000, PTFE vessels, Anton Paar, Graz, Austria. Program: pressurized to 40 bar with N_2_, heated up to 250°C in 20 minutes, held for 20 minutes (max. pressure: ca. 100 bar)
- ICP-MS: 7900, Agilent Technologies, Tokyo, Japan
- ICP-MS/MS: 8800, Agilent Technologies, Tokyo, Japan, with a Micromist nebulizer and a Scott-type spray chamber. Operated in O_2_-mode with a 30% 4^th^ cell gas flow rate (pure O_2_, corresponding to 0.3 mL min^-1^).
- Laser ablation: Iridia laser ablation system, Teledyne Photon Machines, Bozeman, MT, USA, equipped with a 193 nm ATLEX-I-LR ArF* excimer-based nanosecond 1 kHz lasing system (MLase AG, Germering, Germany) and a Cobalt ablation chamber with optimized cup-type ablation cell, connected to an Agilent 7900 ICP-mass spectrometer via PEEK tubing (0.75 mm diameter, 520 mm length) and the Aerosol Rapid Introduction System (ARIS). Helium was used as carrier gas, to transport the generated aerosol from the LA unit to the ICP-MS, and was mixed in the ARIS with Ar make-up gas.
- Cryostat: Thermo Shandon FSE, Waltham, MA, USA

# ICP-MS/MS and LA-ICP-MS measurements

**Table S1.** Settings and typical sensitivity of ICP-MS/MS measurements for determining bulk element concentrations. Oxygen reaction mode (30% 4^th^ cell gas flow).

| **Element** | **m/z** | **Sensitivity [kCPS/1 µg L^-1^]** |
| --- | --- | --- |
| Cu | 63 🡪 63 | 30 |
| Se (🡪 SeO^+^) | 80 🡪 96 | 1.7 |
| Ag | 107 🡪 107 | 80 |
| Hg | 202 🡪 202 | 13 |
| Ge (internal standard) | 74 🡪 74 | 4.8 |
| Lu (internal standard) | 175 🡪 175 | 0.7 |

**Table S2.** Optimized performance data of LA-ICP-MS measurements. Optimization carried out while ablating NIST SRM 610 (Fluence: 4.00 J cm^-2^, Rep. rate: 50 Hz, Dosage: 20, Spot size and shape: 20 µm circle)

| **parameter** | **m/z** | **Signal for NIST 610 [kCPS]** |
| --- | --- | --- |
| U^+^ | 238 | ~ 1600 |
| Th^+^ | 232 | ~ 1500 |
| UO^+^/U^+^ | 254/238 | < 0.3% |
| U^+^/Th^+^ | 238/232 | ~ 107% |

# Optimization of preparation procedures for calibration standards


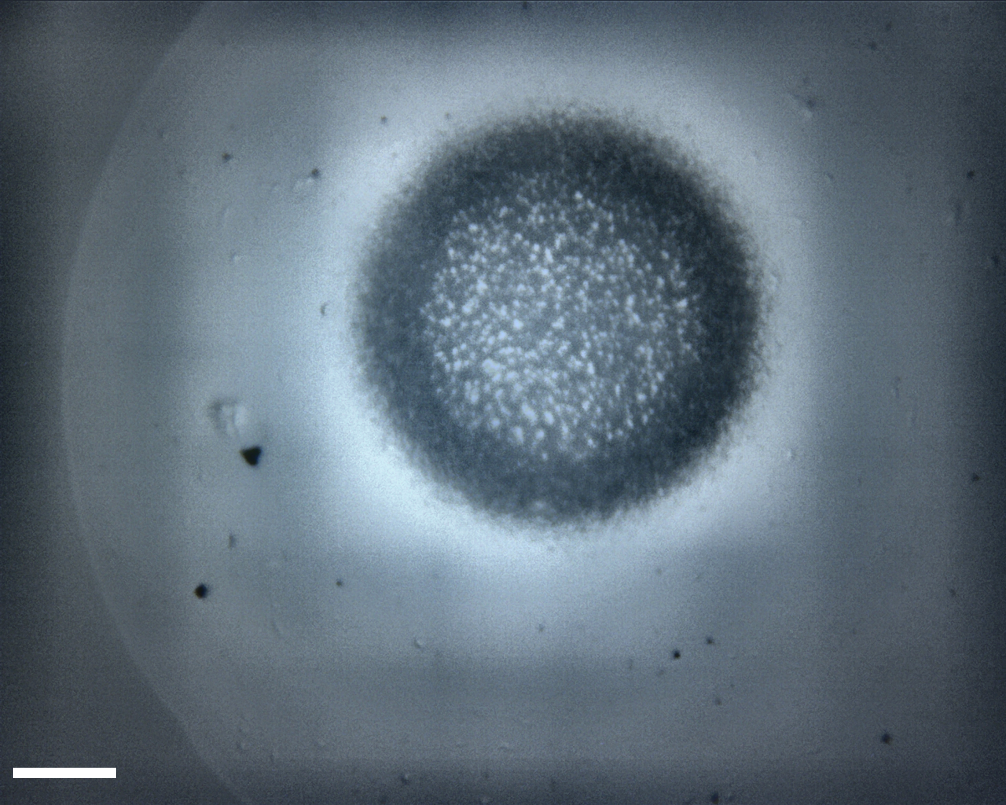


**Fig. S1**. Microscopic image of a droplet containing 1% (m/m) chitosan and 1% (m/m) gelatin. Length of white scale bar: 250 µm.


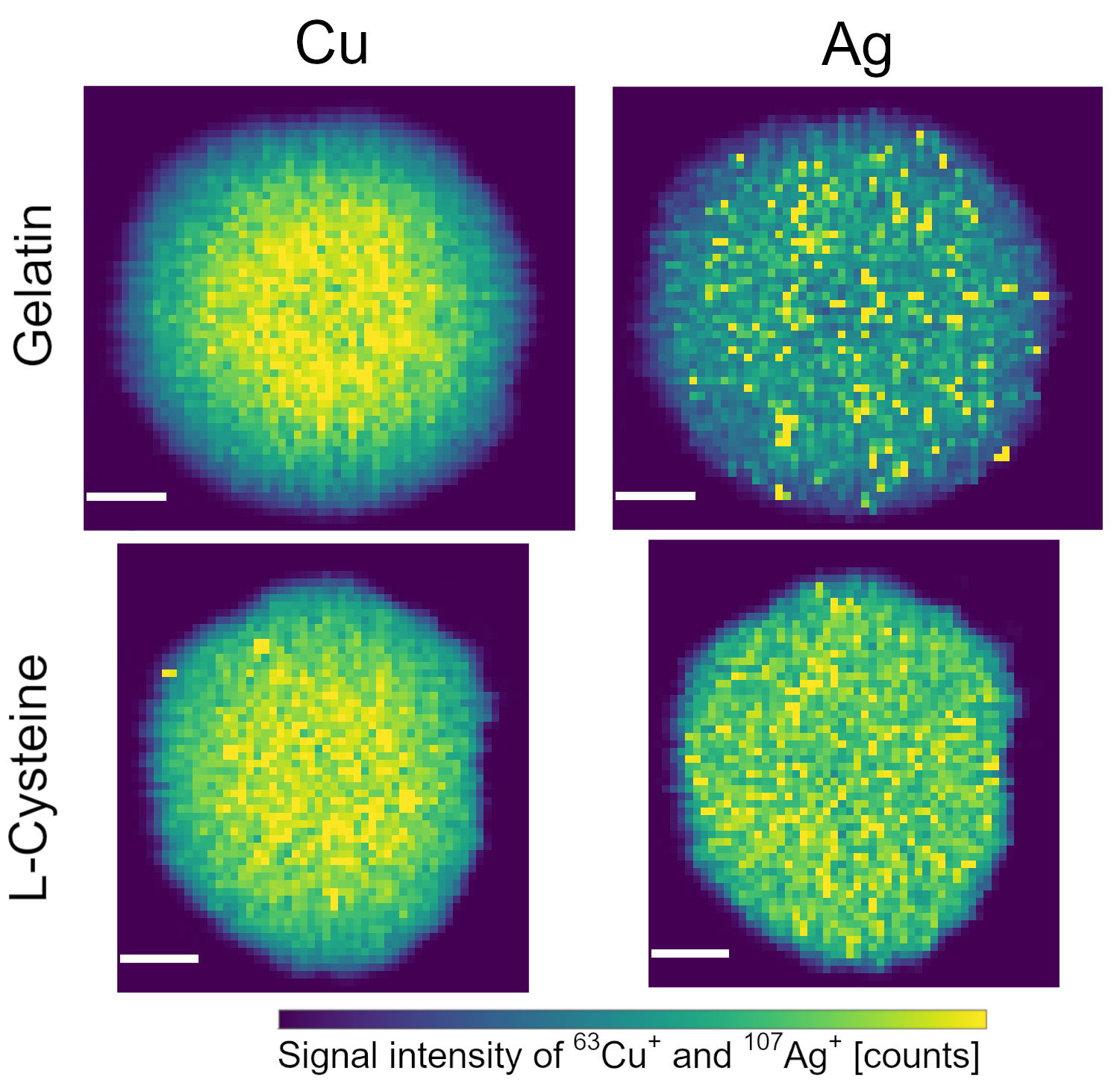


**Fig. S2**. Distribution of Cu and Ag in 10% gelatin (“Gelatin”) and 10% gelatin + 5 mmol L^-1^ L-cysteine (“L-cysteine”) micro-droplets, containing 5 µg g^-1^ Cu and Ag (and 5 µg g^-1^ Hg and 25  µg g^-1^ Se, shown in main article, Fig. 3). The minimum-maximum signal intensities for the color bars are 0-9*10^4^ (Cu, gelatin), 0-7.3*10^4^ (Cu, L-cysteine), 0-5.5*10^4^ (Ag, gelatin) and 0-4.2*10^4^ (Ag, L-cysteine) counts. All white scale bars are 200 µm.


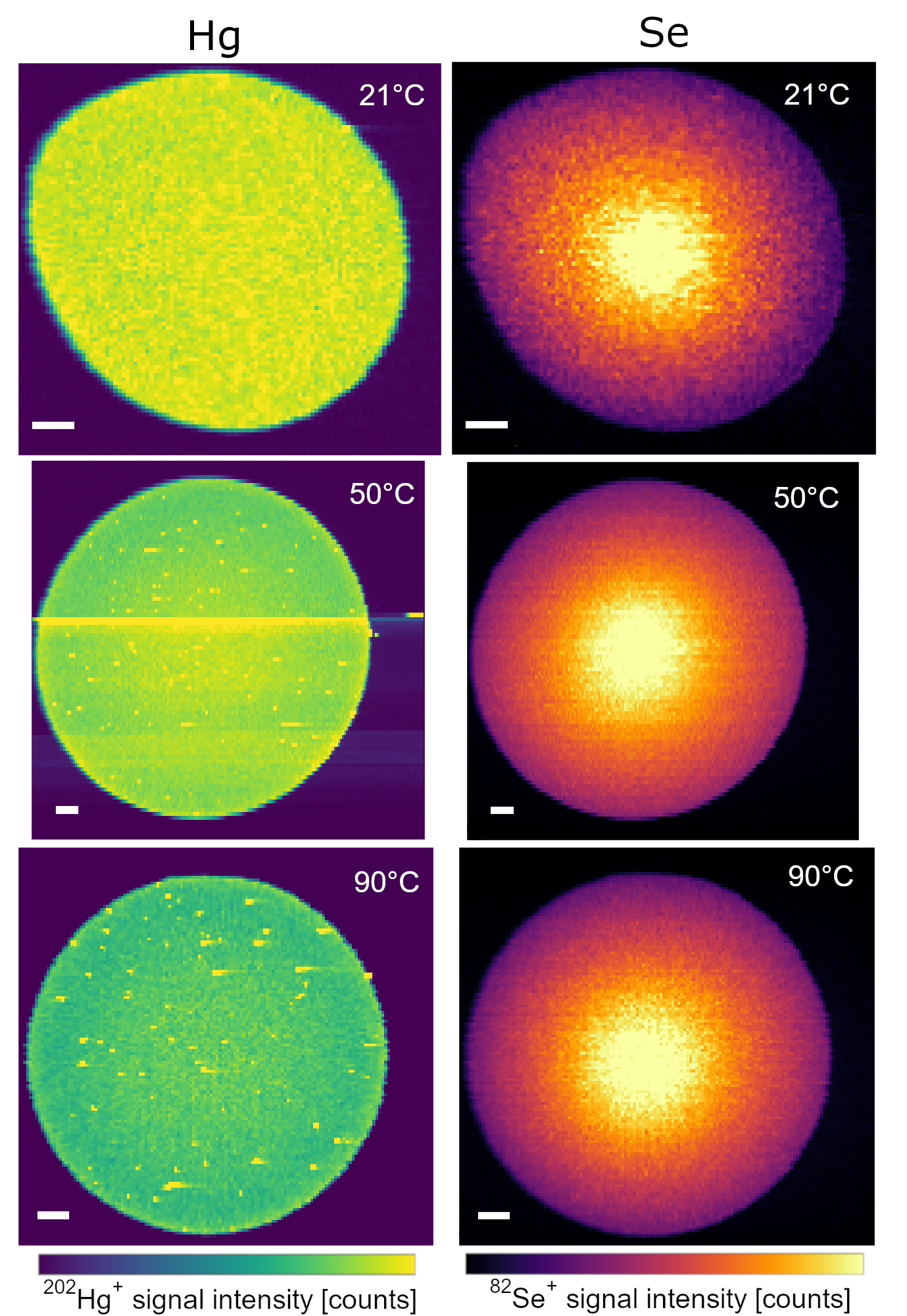


**Fig. S3**. Distribution of Hg (5 µg g^-1^, left) and Se (25 µg g^-1^, right) in 10% gelatin droplets after drying at different temperatures (top: 21°C, middle: 50°C, bottom: 90°C). The minimum-maximum signal intensities for the color bars are 0-0.5*10^3^, 0-4*10^3^ and 0-3*10^3^ counts for ^202^Hg^+^ and 0-0.18*10^3^, 0-1.2*10^3^ and 0-0.8*10^3^ counts for ^82^Se^+^, from top to bottom, respectively. All white scale bars (bottom left corners) are 200 µm.





**Fig. S4**. Calibration of Hg with the optimized preparation procedure. The grey area shows the 95% confidence interval of the linear regression. -



**Fig. S5**. Calibration of Se with the optimized preparation procedure. Th grey area shows the 95% confidence interval of the linear regression. -

# Hg and Se images of mushroom fruitbody samples


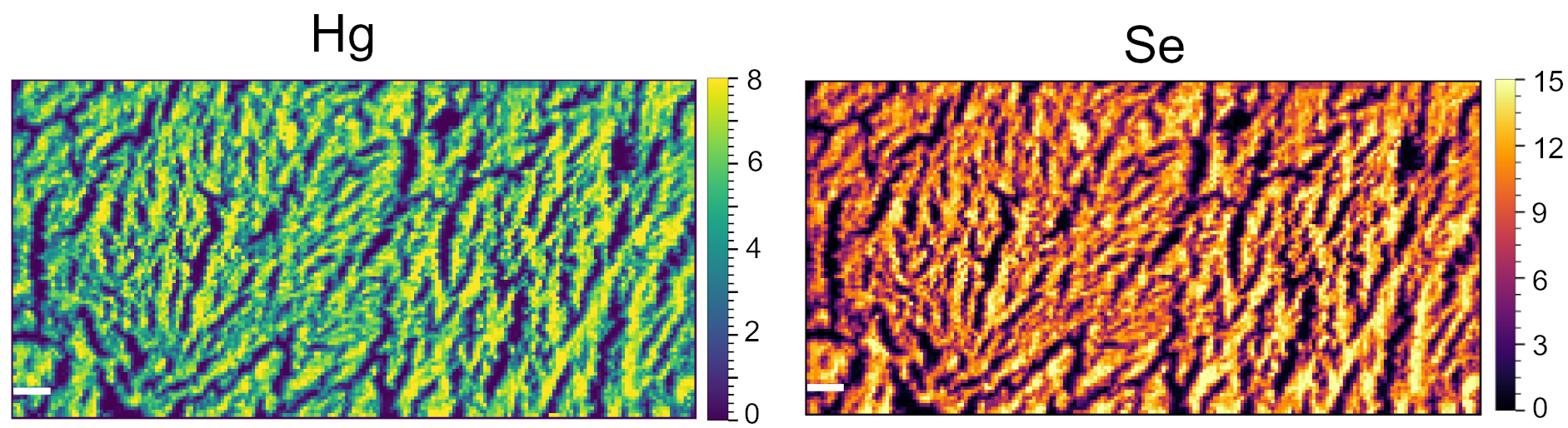


**Fig. S6**. Hg and Se distribution in the in-house reference material MuRM (*Boletus edulis*). Measured nuclides: ^202^Hg^+^ and ^82^Se^+.^ Unit of scale bars: µg g^-1^. Length of white scale bars: 200 µm. The image was put together from 5 separate measurements of 0.4*4 mm size.


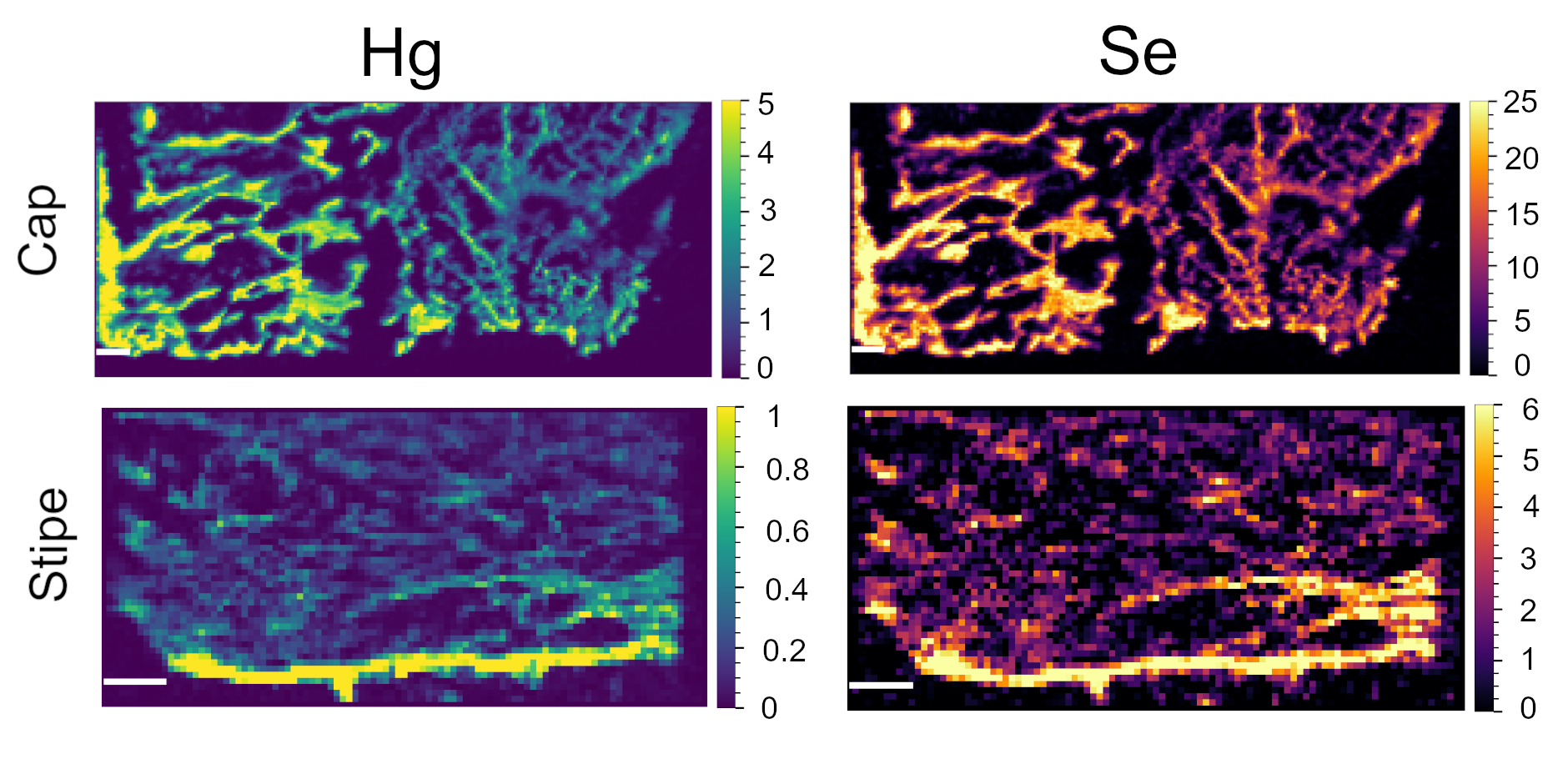


**Fig. S7**. Hg and Se distribution in thin sections of the cap, including tubes (tubes on the left side, cap context on the right side of the maps) and of the stipe (peripheral tissue at the bottom of the maps, context above) of POR-025 (*Boletus aereus*). Unit of scale bars: µg g^-1^. Length of white scale bars: 200 µm.


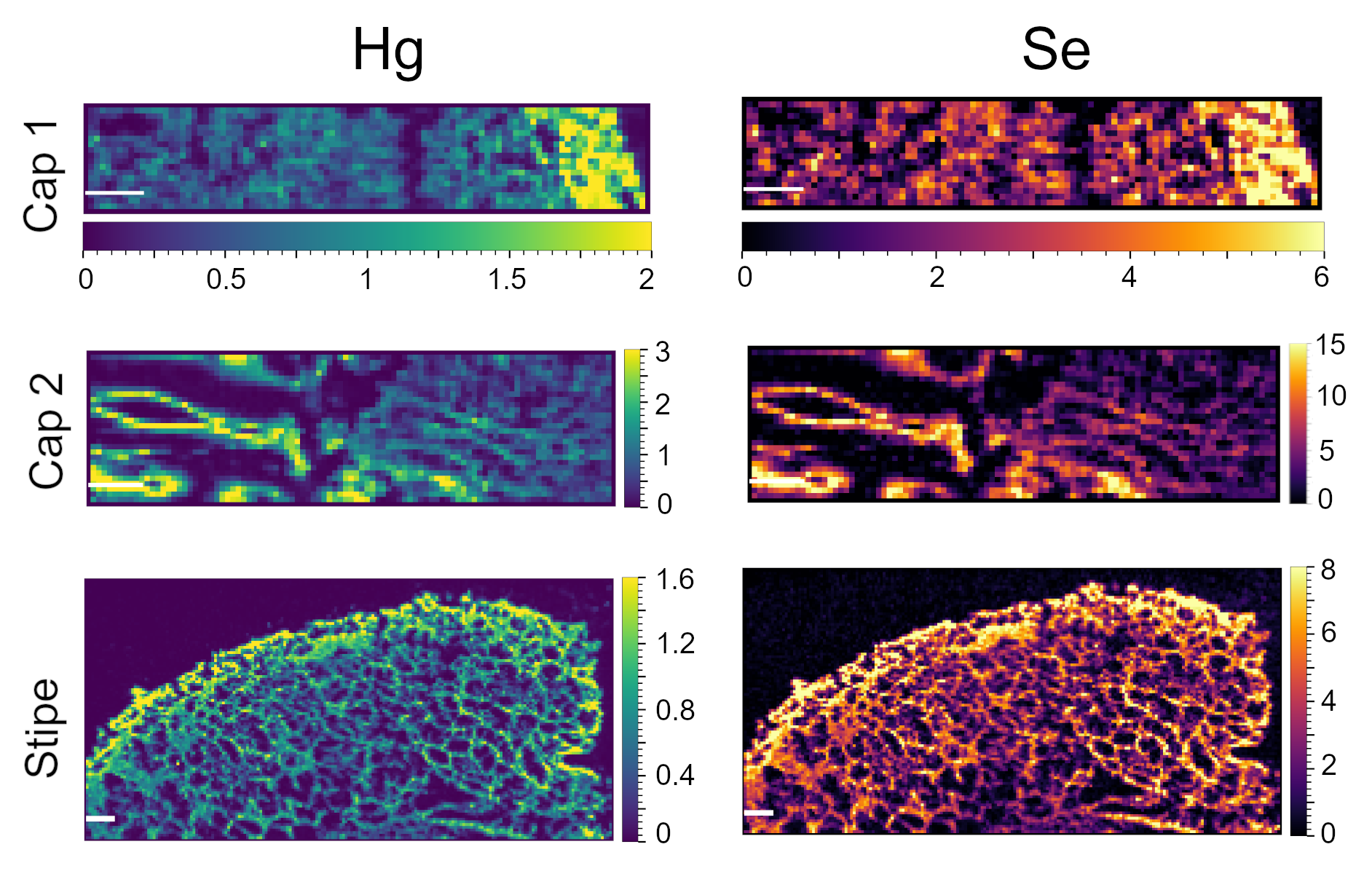


**Fig. S8**. Hg and Se distribution in thin sections of the cap (Cap 1: peripheral tissue on the right side of the maps, cap context on the left side of the maps, cap 2: tubes on the left side, context tissue on the right side of the maps) and of the stipe (peripheral tissue at the top of the maps, context below) of POR-026 (*Boletus aereus*). Unit of scale bars: µg g^-1^. Length of white scale bars: 200 µm.


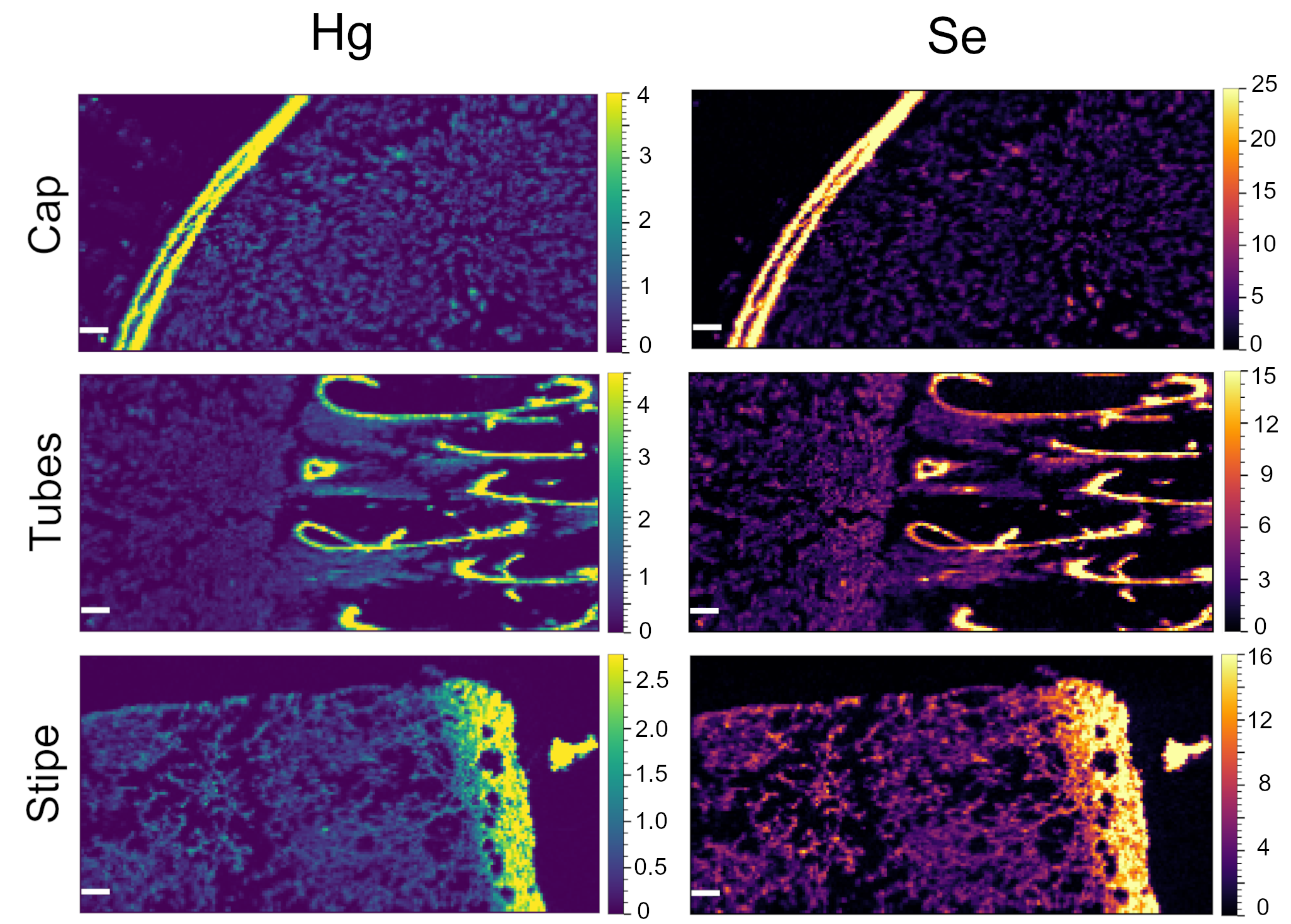


**Fig. S9**. Quantitative distribution of Hg (left) and Se (right) in 4 × 2 mm areas of thin sections of sample POR-031 (*Boletus pinophilus*), color scales µg g^-1^. Top: Cap (peripheral tissue on the left side, similar to location A in Fig. 1). Middle: Tubes (incl. adjacent cap tissue on the left side, similar to location B in Fig. 1). Bottom: Stipe (peripheral tissue on the right side, similar to location C in Fig. 1). All white scale bars are 200 µm. This is the same as Fig. 4 (main article), and added here for completeness.


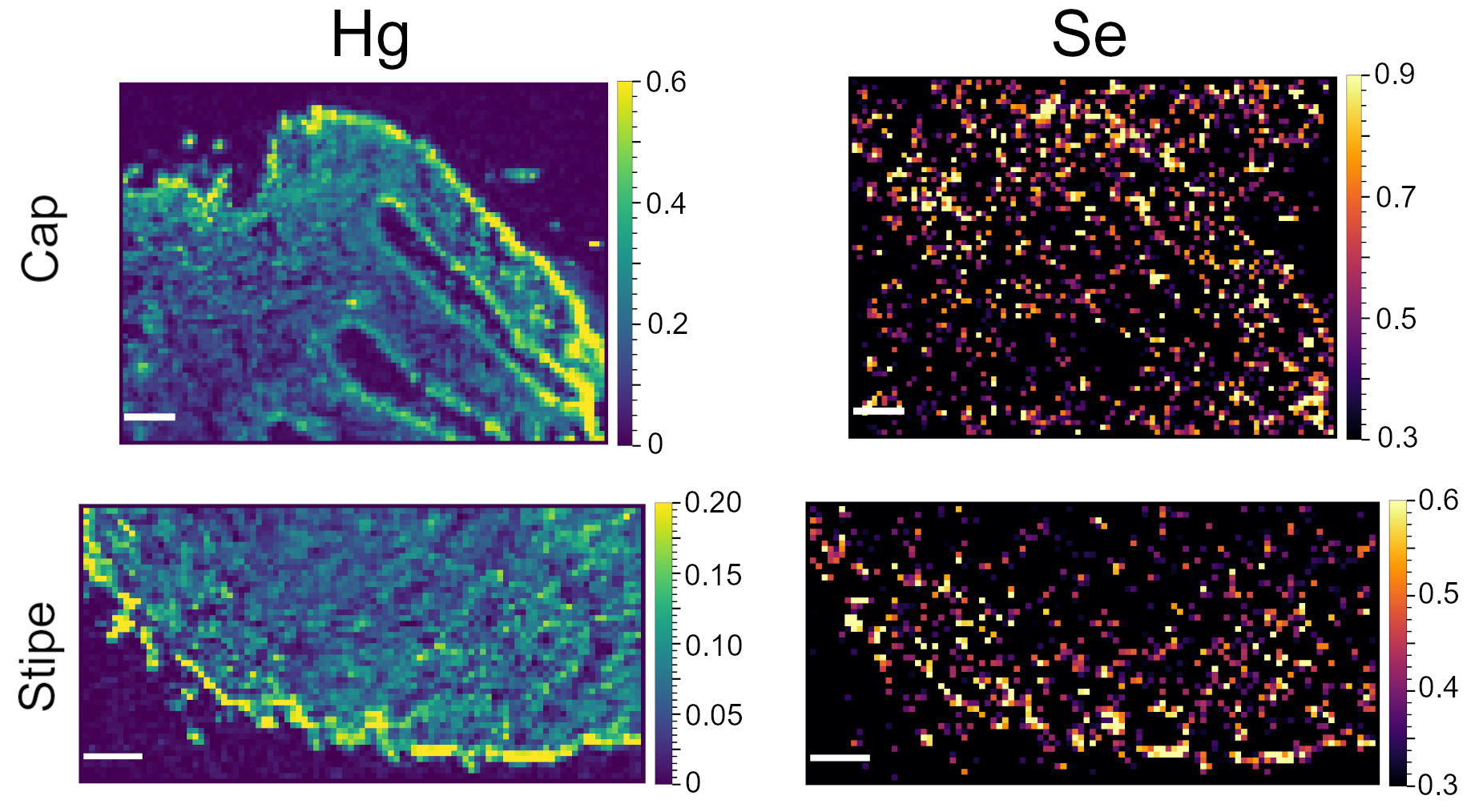


**Fig. S10**. Hg and Se distribution in thin sections of the cap, including lamellae (lamellae on the right side, cap context on the left side of the images), and of the stipe (peripheral tissue at the bottom of the images) of POR-067 (*Macrolepiota procera*). Unit of scale bars: µg g^-1^. Note that the scales of Se only start at 0.3 (=LOD). Length of white scale bars: 200 µm.

**
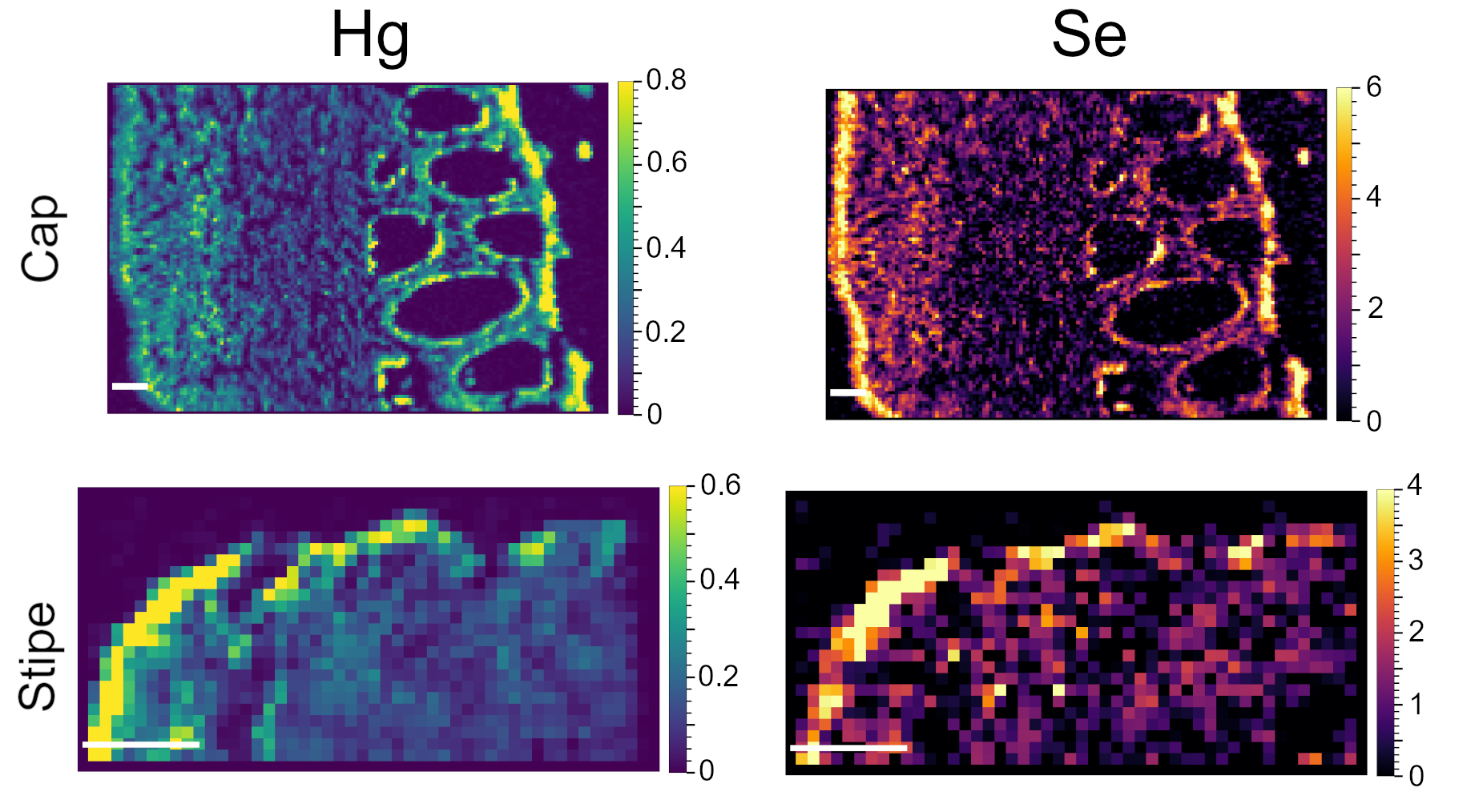
**

**Fig. S11**. Hg and Se distribution in thin sections of the cap (including tubes on the right side, cap context and peripheral tissue on the left side of the images), and of the stipe (peripheral tissue on the left and upper part of the images) of POR-070 (*Boletus edulis*). Unit of scale bars: µg g^-1^. Length of white scale bars: 200 µm.


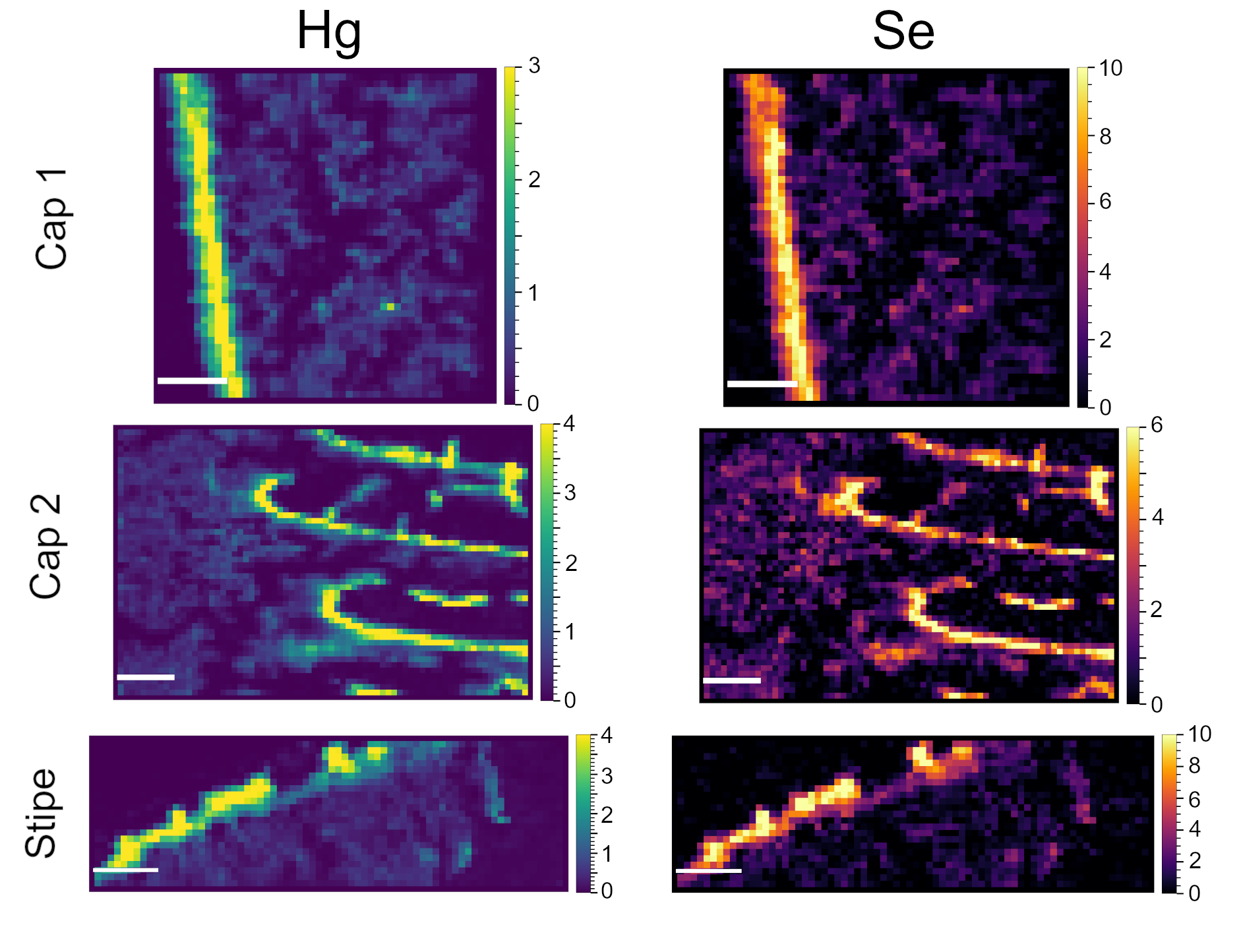


**Fig. S12**. Hg and Se distribution in thin sections of cap (Cap 1: peripheral tissue on the left side of the maps, context tissue on the right. Cap 2: Tubes on the right side of the maps, context tissue on the left), and of the stipe (peripheral tissue on the left and upper part of the maps) of STM-394 (*Boletus edulis*). Unit of scale bars: µg g^-1^. Length of white scale bars: 200 µm.
